# A novel aminopeptidase N/CD13 inhibitor selectively targets an endothelial form of CD13 after coupling to proteins

**DOI:** 10.1007/s00018-023-05102-1

**Published:** 2024-01-30

**Authors:** Giulia Anderluzzi, Michela Ghitti, Anna Maria Gasparri, Giulia Taiè, Angelina Sacchi, Alessandro Gori, Annapaola Andolfo, Federica Pozzi, Giovanna Musco, Flavio Curnis, Angelo Corti

**Affiliations:** 1 Tumor Biology and Vascular Targeting Unit, Division of Experimental Oncology, IRCCS San Raffaele Scientific Institute, Via Olgettina 58, 20132 Milan, Italy; 2 Università Vita-Salute San Raffaele, Milan, Italy; 3 Biomolecular NMR Group, Division of Genetics and Cell Biology, IRCCS Ospedale San Raffaele Scientific Institute, Milan, Italy; 4 Istituto di Scienze e Tecnologie Chimiche, C.N.R., Milan, Italy; 5 ProMeFa, Proteomics and Metabolomics Facility, Center for Omics Sciences, IRCCS Ospedale San Raffaele Scientific Institute, Milan, Italy

**Keywords:** Enzyme inhibitor, Aminopeptidase N, CD13, Endothelial cells, Angiogenesis, Tumor therapy, Tumor necrosis factor-alpha

## Abstract

Aminopeptidase N/CD13, a membrane-bound enzyme upregulated in tumor vasculature and involved in angiogenesis, can be used as a receptor for the targeted delivery of drugs to tumors through ligand-directed targeting approaches. We describe a novel peptide ligand (VGCARRYCS, called “G4”) that recognizes CD13 with high affinity and selectivity. Enzymological and computational studies showed that G4 is a competitive inhibitor that binds to the catalytic pocket of CD13 through its N-terminal region. Fusing the peptide C-terminus to tumor necrosis factor-alpha (TNF) or coupling it to a biotin/avidin complex causes loss of binding and inhibitory activity against different forms of CD13, including natural or recombinant ectoenzyme and a membrane form expressed by HL60 promyelocytic leukemia cells (likely due to steric hindrance), but not binding to a membrane form of CD13 expressed by endothelial cells (ECs). Furthermore, G4-TNF systemically administered to tumor-bearing mice exerted anticancer effects through a CD13-targeting mechanism, indicating the presence of a CD13 form in tumor vessels with an accessible binding site. Biochemical studies showed that most CD13 molecules expressed on the surface of ECs are catalytically inactive. Other functional assays showed that these molecules can promote endothelial cell adhesion to plates coated with G4-avidin complexes, suggesting that the endothelial form of CD13 can exert catalytically independent biological functions. In conclusion, ECs express a catalytically inactive form of CD13 characterized by an accessible conformation that can be selectively targeted by G4-protein conjugates. This form of CD13 may represent a specific target receptor for ligand-directed targeted delivery of therapeutics to tumors.

**Supplementary Information:**

The online version contains supplementary material available at 10.1007/s00018-023-05102-1.

## Introduction

A large amount of work has been done to identify molecules capable of interacting with receptors expressed by the tumor endothelium in an attempt to obtain new drugs capable of modulating the physiology of the tumor vasculature and/or new ligands useful for the targeted delivery of drugs to tumors. Among the various receptors identified to date, aminopeptidase N (APN, EC 3.4.11.2, also referred to as CD13) has attracted the interest of many investigators, owing to the fact that this membrane-bound enzyme is upregulated in the tumor vasculature, as well as to its role in angiogenesis and tumor growth [1–5].

CD13 is a 150–240 kDa zinc metallopeptidase consisting of an enzymatic extracellular domain, transmembrane region, and short cytoplasmic tail, which catalyzes the cleavage of neutral or basic N-terminal amino acid residues of various peptides and proteins [6], with a preference for Met, Ala, Leu, Arg, Tyr, Trp, Phe, Lys, Gln, lle, His, and Val (in order of decreasing efficiency), whereas Pro and acidic amino acid residues are not favored [7]. As part of the M1-family of metalloenzymes, CD13 contains the H_388_EXXH_392_ Zn-binding motif and the G_352_XMEN_356_ catalytic motif (where *X* is any amino acid) within the catalytic site [8]. It has been proposed that, like other M1-family members, CD13 can adopt open and closed conformations relevant to the catalytic mechanism: while the substrate has access to the active site in the open state, its hydrolysis occurs in the closed state [9–11].

Substantial experimental evidence suggests that this enzyme regulates cell adhesion, differentiation, proliferation, migration, and apoptosis in normal and neoplastic tissues, as well as inflammation, antigen presentation, viral infection, and hormone/cytokine activity [12]. In addition to its role in cancer, this enzyme also plays a role in the pathogenesis of myocardial infarction, rheumatoid arthritis, and other inflammatory diseases [12–16]. In tumors, CD13 is overexpressed by endothelial cells and pericytes and, in some cases, by fibroblasts and tumor cells [12, 16, 17]. CD13 is also expressed by many healthy tissue cells, such as epithelial cells of the renal proximal tubules, small intestine, bile duct canaliculi, and prostate, as well as by several other cell types, including mast cells, monocytes, macrophages, granulocytes, and their progenitors [12, 16, 18, 19]. Interestingly, compounds coupled to peptides containing the NGR sequence, a CD13 recognition motif, can bind CD13-positive tumor blood vessels, but not other CD13-rich normal tissues [2, 20], suggesting that different forms of this enzyme are expressed in tumors and normal tissues. Because of this property, peptides containing the NGR motif have been used by many investigators as tumor-homing ligands for the targeted delivery of chemotherapeutic drugs, liposomes, antiangiogenic compounds, DNA complexes, viral particles, and cytokines to tumors with improved therapeutic efficacy [21]. Notably, one of these compounds, NGR-TNF (consisting of CNGRCG peptide fused to tumor necrosis factor-alpha), has been tested in patients with relapsing/refractory primary central nervous system lymphoma (PCNSL), with evidence of antitumor efficacy and good tolerability [20, 22, 23]. Results obtained from preclinical and clinical studies of NGR-TNF and other NGR-drug conjugates highlight the value of the tumor-associated form of CD13 as a target receptor for the ligand-directed targeted delivery of drugs to tumors. However, the molecular basis of the selective recognition of CD13 expressed in neoplastic tissues by NGR-drug conjugates is still unknown.

The present study was undertaken to identify new ligands of CD13 and elucidate the structural basis of the selective recognition of CD13 in tumors by peptide–drug conjugates. In preliminary studies performed with control peptides, we serendipitously discovered a novel peptide ligand of CD13, characterized by the VGCGRRYCSN sequence, capable of interacting with the active site of recombinant soluble CD13 and inhibiting its catalytic activity. We show here that an optimized version of this peptide (VGCARRYCS, disulfide-bridged, called “G4”) can bind and block the active site of CD13 with high affinity and selectivity, but not after fusion to TNF or after coupling to other bulky compounds; nevertheless, we also show that the G4-TNF fusion protein can still target tumor lesions, when administered to tumor-bearing mice, through a CD13-dependent mechanism. Using computational, biochemical, and biological methods, we provide experimental evidence to suggest that G4-TNF can recognize a catalytically inactive endothelial form of CD13 characterized by an open and accessible binding site, but not the catalytically active CD13; the latter has a binding pocket accessible only to the free G4 peptide.

## Materials and methods

### Cell lines and reagents

Human umbilical vein endothelial cells (HUVEC, ATCC, CRL-1730) were cultured in endothelial cell growth medium-2 (Lonza). HL-60 promyeloblasts (ATCC, CCL-240) were cultured in RPMI medium supplemented with 10% fetal bovine serum, 2 mM glutamine, 50 µg/mL streptomycin, and 100 U/mL penicillin. BL21 Star (DE3) *E.coli* cells (cat. C601003, Thermo Fisher). Dulbecco’s phosphate-buffered saline without Ca^++^ and Mg^++^ (DPBS) (EuroClone), human serum albumin (HSA) (Baxter), bovine serum albumin (BSA, cat. A2153), normal human serum type AB (cat. 14490e, BioWhittaker), water for injection (SALF). Bestatin-hydrochloride (cat. B8385-5MG), l-alanine-p-nitroanilide hydrochloride (cat. A9325) were obtained from Sigma-Aldrich. Phenyl-Sepharose 6 Fast Flow (cat. 17097305), DEAE-Sepharose Fast Flow (cat. 17070901), and Sephacryl-S 300 HR (cat. 17059901) were obtained from Cytiva. l-alanine 7-amido-4-methylcoumarin (cat. 4028736), l-glutamic acid *γ-*(7-amido-4-methylcoumarin) (cat. 4002702), and l-arginine-7-amido-4-methylcoumarin (cat. 4002148) were obtained from Bachem; His-tagged recombinant human CD13 (rCD13) (cat. 10051-H08H) consisting of the CD13 extracellular domain expressed in HEK-293 cells, His-tagged recombinant human aminopeptidase A (APA) (cat. 10554-H07B) and rabbit anti-human CD13 polyclonal antibody (cat. 10051-RP02) were purchased from Sino Biological. His-tagged recombinant human aminopeptidase B (APB) (cat. 8089-ZN-010, Bio-Techne), anti-human CD13 monoclonal antibody (mAb) WM15 (Cat. 301708, BioLegend), anti-mouse CD13 mAb R3-63 (cat. MA516931, Invitrogen), FITC-labeled goat anti-mouse IgG (H + L) secondary antibody (cat. A16067, Life Technologies); Pierce BCA Protein Assay Kit (cat. 23227, Thermo Fisher); anti-β-actin monoclonal antibody (cat. 1978, Sigma).

### Peptide synthesis and cyclization

The peptides used in this study were synthesized, cyclized, purified, and analyzed by mass spectrometry and reverse-phase HPLC as described in Supplementary Methods. The source, the molecular mass, and the purity of each peptide are reported in Supplementary Table S1. The mass spectra and RP-HPLC chromatograms of the VGCARRYCS peptide Lot#A (synthetized and analyzed by Proteogenix) are shown in Supplementary Fig. S1.

### Enzyme activity and inhibition assays of recombinant CD13

Recombinant CD13 (rCD13) enzyme activity and inhibition assays were performed as described previously using l-alanine-p-nitroanilide [24] or l-alanine 7-amido-4-methylcoumarin as substrates in 60 mM Tris–HCl, pH 7.4, or 60 mM potassium phosphate buffer, pH 7.4. Mixtures containing peptides at various concentrations, rCD13 (0.2 µg/mL), and l-alanine-p-nitroanilide substrate (0.5 mM) in 60 mM potassium phosphate buffer, pH 7.4 (total volume, 0.1 ml) were prepared and left to react for 30 min at 37 °C. The products were then analyzed using a plate reader (iMark Microplate Absorbance Reader; Bio-Rad). Alternatively, the assay was performed using 0.1 mM l-alanine 7-amido-4-methylcoumarin in 60 mM Tris–HCl, pH 7.4, as a substrate. Fluorescence intensity of methylcoumarin was measured on an Infinite 200 PRO plate reader (Tecan) using the following settings: *excitation wavelength*: 341 nm; *emission wavelength*: 441 nm; *excitation bandwidth*: 9; *emission bandwidth*: 20 nm; *gain*: 109 manual; *number of flashes*: 25; *integration time*: 20 µs; *lag time*: 0 µs; *settle time*: 0 ms.

The IC_50_ of each inhibitor was calculated by non-linear regression analysis of inhibition data using GraphPad Prism Software (GraphPad Software, Version 9.00, San Diego, CA). Steady-state enzyme kinetic analyses were performed using various concentrations of l-alanine-p-nitroanilide (0–1.5 mM) and VGCARRYCS peptide (0–900 nM). The initial velocities (*Vo*) were calculated from the slopes of the first 5 min of reaction. The enzyme inhibitory constant (*Ki)* was calculated using the competitive enzyme inhibition model in GraphPad Prism Software.

### Enzymatic inhibition assay of natural sCD13

The effect of peptides on the enzymatic activity of natural soluble CD13 from human plasma (sCD13) was assessed as follows: 96-well polyvinyl chloride microtiter plates were coated with anti-CD13 mAb WM15 (5 µg/mL in DPBS, 50 μL/well, overnight at 4 °C). All subsequent steps were performed at room temperature. The plate was washed with DPBS and further incubated with DPBS containing 3% BSA (200 μL/well; 1 h). Normal human serum was diluted 1:2 vol/vol with DPBS containing 2% BSA and then added to a previously washed plate (100 μL/well; 90 min to allow CD13 binding). After washing with DPBS, the plate was filled with a mixture containing serial dilutions of peptides and l-alanine-p-nitroanilide (4.5 mM, 100 μL/well in 60 mM Tris–HCl, pH 7.4). After 2 h of incubation at 37 °C, the absorbance at 405 nm of the p-nitroanilide product was quantified using a plate reader (iMark Microplate Absorbance Reader, Bio-Rad).

### Enzyme activity assays of recombinant APA and APB

The effect of peptides on the enzymatic activity of APA and APB was evaluated using the method described previously [24] with some modifications. Briefly, threefold serial dilution of peptides (ranging from 0 to 10 or 100 µM) were incubated with either APA (0.2 µg/mL in 60 mM Tris–HCl pH 7.4, 50 mM CaCl_2_, 200 mM NaCl) or APB (0.2 µg/mL in 60 mM Tris–HCl pH 7.4, 100 mM KCl) and relative substrates at 0.1 mM (l-glutamic acid *γ*-(7-amido-4-methylcoumarin) or l-arginine-7-amido-4-methylcoumarin, respectively) in 96-well bottom clear black polystyrene microtiter plates (100 µl/well). The mixture was incubated for 30 min at 37 °C in a humid box, and the cleavage of the substrate was correlated with aminopeptidase enzymatic activities by measuring the fluorescence of methylcoumarin at *λ*_ex_ 341 nm and *λ*_em_ 441 nm with a plate reader (Infinite 200 PRO, Tecan). IC_50_ was calculated as reported above for CD13 activity.

### Competitive binding assays of anti-CD13 mAb WM15 to HUVEC with G4 by flow cytometry

HUVEC were seeded in 96-well U-bottom polystyrene microtiter plates at a density of 2 × 10^5^ cells in 100 µL DPBS supplemented with 5% normal human serum (NHS) and mixed with a mixture of peptide G4 ranging from 0 to 10 µM and mAb WM15 (0 or 0.13 nM). The chromogranin A_429-439_ peptide fragment (CgA_429-439_) was used instead of G4 as a control. After 1 h of incubation at 4 °C, the cells were washed and resuspended in 100 µL of DPBS containing 5% NHS and 1 µg/mL of FITC-labeled goat anti-mouse IgG secondary antibody. Cells were subsequently incubated for 1 h at 4 °C, washed, and analyzed by flow cytometry. All flow cytometry data were collected on a CytoFLEX S flow cytometer (Beckman Coulter) and analyzed using FlowJo software 10 (BD BioScience).

### Production and characterization of G4-TNF fusion protein

Recombinant murine VGCARRYCS-TNF (named G4-TNF) was produced by recombinant DNA technology using the pET-11b expression system (Novagen) in BL21 Star (DE3) *E.coli* cells. G4-TNF was purified from bacterial lysates by ammonium sulfate precipitation, hydrophobic interaction chromatography on Phenyl–Sepharose 6 Fast Flow, ion exchange chromatography on DEAE-Sepharose Fast Flow, and gel-filtration chromatography on Sephacryl-S-300 HR. All the solutions used in the chromatographic steps were prepared using sterile endotoxin-free water. About 4.5 mg of G4-TNF was recovered from 2 L of culture. The protein content of the final product was measured by BCA protein assay kit. The molecular weight of G4-TNF was determined using SDS-PAGE and mass spectrometry. The cytotoxic effects of G4-TNF on L-M fibroblasts were evaluated as previously described [20].

### Antitumor activity of G4-TNF in WEHI-164 fibrosarcoma-bearing mice

Female BALB/c mice (Charles River Laboratories, Calco, Italy), weighing 16–18 g, were subcutaneously injected into the left flank of 10^6^ WEHI-164 cells (ATCC, cat. CRL-1751, Lot. 63,682,839). Dose-finding experiments were performed with groups of six mice treated with G4-TNF (4, 20, or 100 pg/dose in 100 μL of 0.9% sodium chloride containing 100 µg/ml HSA, intraperitoneally injected on day 5) or vehicle. Tumor growth was monitored daily by measuring tumor volume with calipers, as previously described [25]. The animals were sacrificed before the tumor diameter reached 1.0–1.5 cm. To test whether the antitumor activity of G4-TNF was mediated by CD13, tumor-bearing mice (6 animals/group) were treated with a single injection of G4-TNF (100 pg/dose, i.p.) in combination with peptide CNGRC (100 µg/dose) or anti-CD13 mAb R3-63 (25 µg/dose, given 1.5 h i.p. before administration of G4-TNF) 5 days after tumor implantation.

### G4/CD13 molecular docking model

Molecular docking experiments were performed using Maestro Schrödinger Suite software (Schrödinger Release 2020-3: Maestro, Schrödinger, LLC, New York, NY, 2021). To evaluate the conformational space of VGCARRYCS, Prime Macrocycle Conformational Sampling (Prime-MCS) [26] was used to generate 996 structures. X-ray structures of human CD13 in closed and intermediate open conformations (PDB codes:4FYR and 5LHD [8, 11]) were prepared using the Protein Preparation Wizard tool [27]. Crystallographic waters and hetero groups were removed, except for the catalytic Zn^2+^ ion; hydrogen atoms were added, and missing side chains and loops were filled. Structures were optimized at pH 7.0 and minimized with OPLS2005 with a root mean square deviation tolerance on heavy atom of 0.5 Å. The grids were centered on Zn^2+^ and the box dimensions were set to fit ligands smaller than 25 Å. The hydroxyl groups in Y477, S897, S899, S415, and T860 were rotated. Decoy poses were generated using the extra-precision mode of Glide [28] by flexible docking. Nitrogen inversion and predefined functional groups were sampled, enhancing conformational sampling by two times. Post-docking minimization was performed by applying a strain correction term to the pose. Docking poses were then filtered according to the presence of an H-bond between V_1_ of the peptide and E_355_ of CD13, which belongs to the G_352_xMEN_356_ substrate-guiding motif. To estimate the relative binding free energies of the selected protein–ligand complex models, we performed a post-process calculation based on molecular mechanics using the generalized born surface area (MM-GBSA) method. The Schrödinger Prime module [29] was used to select the OPLS4 force field and the VSGB model. We considered all protein residues within 3.5 Å from the ligand. The best poses in terms of the MM-GBSA binding energies were selected.

The docking model of G4 in complex with human CD13 in the intermediate open form was used to generate a graphical representation of the G4-TNF fused protein in complex with CD13. TNF residues ranging from 77 to 89, which belong to an unfolded region of the protein, were built in an extended conformation and minimized using Maestro Schrӧdinger Suite. One unit of the trimeric crystallographic TNF_89-233_ (PDB code:6RMJ) structure was then linked to VGCARRYCS-TNF_77-89_ by superimposing the backbone of V89_TNF89-233_ to the backbone of V89_VGCARRYCS-TNF77-89_. The solvent-accessible surface area (SASA) was calculated using the PISA server [30]. Molecular images were generated using the Maestro Schrӧdinger Suite and the PyMOL open version [http://www.pymol.org/pymol].

### Quantification of CD13 expressed by HUVEC and HL-60 cells by western blotting

HUVEC or HL-60 cells (15 × 10^6^ cells) were centrifuged and incubated with 300 µL of lysis buffer consisting of 100 mM octyl β-D-glucopyranoside and 1 mM phenylmethylsulfonyl fluoride in DPBS, and then incubated on ice for 30 min. Cell lysates were then centrifuged (16000 × g, 20 min, 4 °C) and the protein concentration in supernatants was measured by BCA protein assay kit. Two micrograms of total proteins were then separated by SDS-PAGE using 12% polyacrylamide gels and analyzed by western blotting using 0.2 µm nitrocellulose membranes (Bio-Rad). The membrane was blocked with 5% milk (Regilait), 0.1% Tween-20 in DPBS (1 h at room temperature). The membrane was then cut with a scalpel at the level of the 50 KDa band of the pre-stained molecular weight marker; the upper part of the membrane with high molecular weight proteins was incubated overnight at 4 °C with a rabbit anti-CD13 polyclonal antibody (1:1000 in DPBS containing 5% milk and 0.1% Tween-20), whereas the lower part was incubated with a mouse anti-β-actin monoclonal antibody (1:1000 in the same buffer). The membranes were then washed with DPBS-0.1% Tween-20 and further incubated for 1 h at room temperature with a horseradish peroxidase-conjugated anti-rabbit immunoglobulin G antibody solution (cat. A4914, Sigma-Aldrich, 1:5000, in DPBS-0.1% Tween-20), or with a horseradish peroxidase-conjugated anti-mouse immunoglobulin G antibody solution (cat. A9044, Sigma-Aldrich, 1:50,000 in DPBS-0.1% Tween-20) for CD13 and actin immunodetection, respectively. After three washing (4 min each) with DPBS-0.1% Tween-20, the antibody binding was detected by chemiluminescence assay using the ECL Prime kit (GE Healthcare) and the ChemiDoc Imaging System (Bio-Rad), and the density of bands corresponding to proteins of about 150 kDa (CD13) and 43 KDa (actin) was quantified using the ImageJ software.

### Cell adhesion assay

The peptide VGCARRYCSGGSGGGSGK-biotin, with a biotin moiety linked to the ε-aminogroup of the Lys residue (ProteoGenix), was complexed with streptavidin (G4-streptavidin) or avidin (G4-avidin) by mixing it with a molar excess of streptavidin or avidin in DPBS (1:6 mol/mol), followed by incubation for 1 h at room temperature under mild stirring. To perform the cell adhesion assay, 96-well polyvinyl chloride microtiter plates were coated with G4-avidin or G4-streptavidin (10 µg/mL in DPBS, overnight, 4 °C). After blocking with 2% BSA in EBM-2 (45 min at room temperature), 4 × 10^5^ HUVEC were seeded in Endothelial Cell Growth Basal Medium-2 (EBM-2, Cat. # 00190860, Lonza) and incubated for 2.5 h at 37 °C, 5% CO_2_. Unbound cells were removed by washing with EBM-2, and adherent cells were fixed with 3% paraformaldehyde and 2% sucrose in DPBS and stained with 0.5% crystal violet. The number of adherent cells was quantified by measuring the absorbance of the wells at 540 nm wavelength using a microplate reader.

## Results

### Identification and optimization of a novel peptide (called G4) that inhibits CD13 enzymatic activity at sub-micromolar concentrations

The inhibitory effect of peptide VGCGRRYCSN (disulfide-bridged) on the catalytic activity of CD13 was tested using mixtures of a) recombinant soluble form of human CD13 ectodomain expressed in HEK-293 cells (rCD13, 0.2 µg/ml), b) l-alanine-p-nitroanilide substrate, and c) VGCGRRYCSN or CSGIGSGGC (negative control peptide) at various concentrations. VGCGRRYCSN inhibited rCD13 with an IC_50_ of 2.01 ± 1.05 µM, whereas no inhibition was observed with CSGIGSGGC (Table 1 and Supplementary Fig. S2A).

**Table 1:**
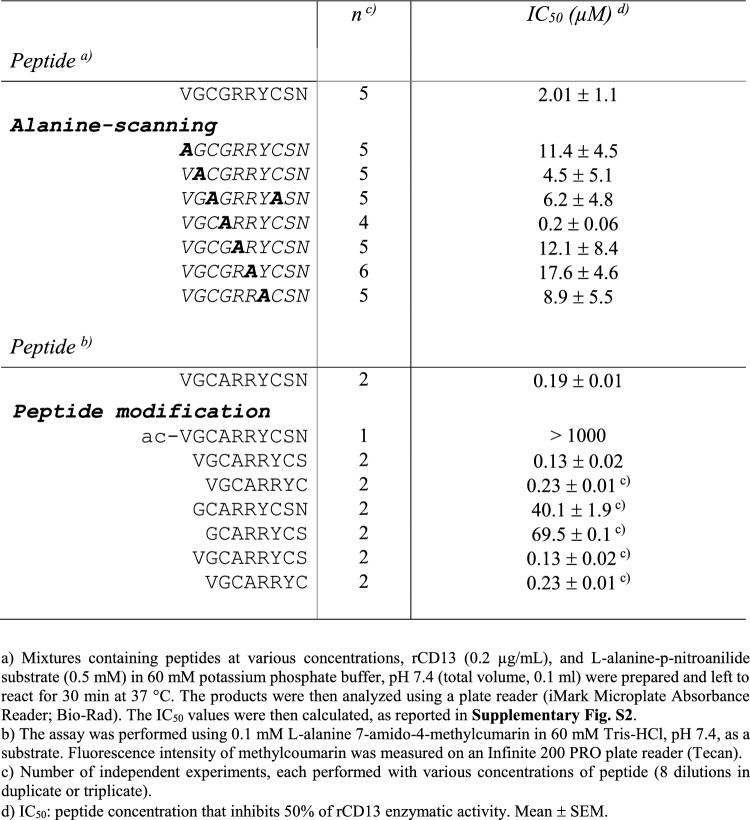
Inhibitory activity of VGCGRRYCSN and its “alanine-scanning” derivatives on rCD13 enzymatic activity

“Alanine-scanning” experiments of VGCGRRYCSN, performed to assess the role of each amino acid residues, showed that the substitution of Val-1, Arg-5, and Arg-6 with Ala reduces five–eightfold its inhibitory activity, while the substitution of Gly-4 increases ~ tenfold its inhibitory effects (IC_50_, 0.20 ± 0.06 µM) (Table 1), suggesting that these residues are important for enzyme recognition. Replacement of Cys-3 and Cys-8 with Ala caused a ~ threefold loss of activity, indicating that the disulfide constraint is also important. Based on these findings, we selected the disulfide-bridged peptide VGCARRYCSN bearing the Gly-4/Ala mutation for subsequent studies.

Other chemical modification studies have shown that N-terminal acetylation of VGCARRYCSN completely abrogates its inhibitory activity (Table 1), as well as its ability to block the binding of rCD13 to mAb WM15, a neutralizing antibody (Supplementary Fig. S2B), indicating that the α-amino group played a crucial role in CD13 recognition.

Deletion of Val-1 caused a 200-fold loss of activity, whereas deletion of Asn-10 did not affect or modestly increased the inhibitory activity (Table 1). Furthermore, deletion of Val-1 and Gly-2 caused a 270-fold loss of activity, whereas deletion of Ser-9 and Asn-10 caused only a threefold decrease. These findings suggest that the N-terminal part of the molecule is crucial for its inhibitory activity, whereas the C-terminal part is less important. Notably, the small disulfide-bridged peptide CARRYC still inhibited rCD13 with an IC_50_ of 51 µM (Table 1). This finding and the observation that replacement of Arg-5 and Arg-6 with Ala in VGCGRRYCSN caused a significant loss of inhibitory activity suggest that the disulfide-constrained loop of the molecule also contributes to CD13 recognition.

In conclusion, these results suggest that the α-amino group of Val-1 and side chains of Arg-5 and Arg-6 play major roles in peptide/CD13 recognition. As Val-1 is crucial for VGCARRYCSN activity, whereas Asn-10 can be deleted with no loss of function, the peptide VGCARRYCS (disulfide-bridged, hereinafter called “G4”) was selected for subsequent experiments.

### The peptide G4 is a competitive inhibitor of rCD13

**Fig. 1:**
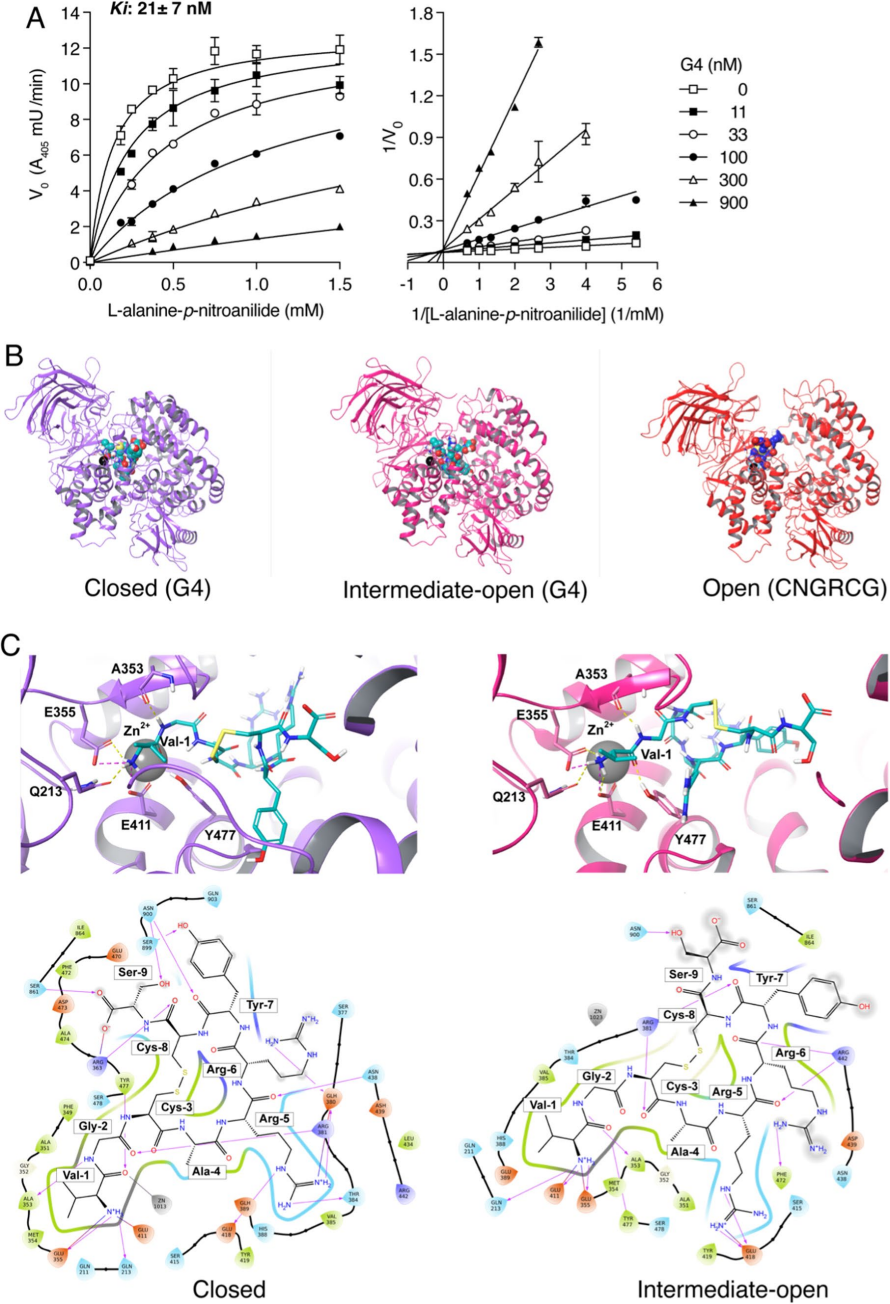
Interaction of peptide G4 with the catalytic site of CD13. **A** Competitive inhibition of rCD13 enzymatic activity by peptide G4. Steady-state kinetic analysis of rCD13 in the presence of different concentrations of l-alanine p-nitroanilide hydrochloride substrate and G4, as indicated, in 60 mM potassium phosphate buffer, pH 7.4, at 37 °C, is shown. The initial velocities (*Vo*) were calculated from the slopes of the first 5 min of reaction. Two independent experiments, each in duplicate, were performed. The results of one representative experiment are shown in the left panel (mean ± SEM). The indicated enzyme inhibitory constant (*K*_*i*_ = 21 ± 7 nM*,* mean ± SEM of two independent experiments) was calculated using the competitive enzyme inhibition model of GraphPad Prism Software. The Lineweaver–Burk plot of the data (right panel) shows that G4 changed the apparent Km of the enzyme but not the Vmax, which is consistent with a competitive mechanism of inhibition. **B** Representative docking poses of G4 in complex with the closed (left) and intermediate open forms of human CD13 (center). For comparison, the crystallographic structure of CNGRCG in complex with the open form of porcine CD13 (pdb code: 4OU3) is shown on the right. CD13 and peptides are represented in cartoon and CPK, respectively. This model suggests that G4 fits into the catalytic pocket of the human CD13. **C** The best docking pose, in terms of ΔG_MMGBSA_, of G4 in complex with the closed and intermediate open form of human CD13 is shown on the left and on the right panels, respectively. CD13 residues involved in electrostatic interactions engaged by Val-1 are highlighted and labeled on the upper panels. The corresponding ligand interaction diagrams are shown in the lower panels. In the diagrams, protein pockets within 4 Å from G4 are displayed as colored spheres, where red, green, purple, and blue indicate acidic, hydrophobic, basic, and polar residues, respectively. H-bonds and salt bridges are shown as lines and arrows. G4 atoms exposed to solvent are highlighted with gray spheres

The location of the binding site of the VGCARRYCS peptide (G4) on CD13 and the mechanism of enzyme inhibition were investigated. Steady-state kinetic analysis of rCD13 in the presence of various amounts of substrate and G4 showed that this peptide could inhibit the enzyme with an inhibition constant (K_i_) of 21 ± 7 nM (Fig. 1A, left). The Lineweaver–Burk double-reciprocal plot of the data showed straight line intercepts converging at a single point on the *y* axis, which is typical of competitive inhibitors (Fig. 1A, right). This finding suggests that G4 interacts with the catalytic site of rCD13.

To assess whether G4 is a substrate that can be cleaved after interaction with rCD13, we analyzed the molecular weight of the peptide after incubation, for various times, with different amounts of rCD13 (0, 0.2, and 2 µg/ml) at 37 °C. Notably, cleavage of Val-1 and Gly-2 was observed only after a long incubation time (16 h) with a large amount of enzyme (2 µg/ml) (Supplementary Fig. S3). This suggests that G4 is a poor substrate of rCD13 and that no structural modifications occurred during the steady-state kinetic analyses performed to determine the Ki value, which were performed with low concentrations of rCD13 and short time intervals. Nevertheless, the observation that G4 was partially cleaved after prolonged incubation with rCD13 at high concentrations confirms the hypothesis that this peptide can interact with the catalytic site of the enzyme.

### G4 can fit into the catalytic pocket of the enzyme in closed and open form

To characterize the interactions between G4 and the catalytic site of CD13 at the molecular level, we investigated whether the peptide could fit inside the catalytic pocket of human CD13 by performing docking calculations. Importantly, CD13 exists in different conformations, named open, intermediate open, and closed forms, based on their active site accessibility, with the open and closed conformations associated with the catalytically inactive and active states, respectively [31]. A comparison of the solvent-accessible surface area (SASA) of the available human CD13 structures (intermediate open and closed forms, PDB code: 5LHD and 4FYR, respectively) showed a Δ_SASA_ of 1581 Å^2^, revealing a drastic reduction in cavity accessibility upon CD13 closure. Therefore, we structurally investigated whether G4 could fit into the more accessible catalytic pocket of the CD13 intermediate open form and into the cavity of the closed state.

Molecular docking experiments performed on the 3D structures of human CD13 showed that G4 can fit into the catalytic pocket of the enzyme in both closed and intermediate open forms, occupying the same region as the CNGRCG peptide previously crystallized in a complex with the open form of porcine CD13 (PDB code 4OU3) [32] (Fig. 1B).

In both the closed and intermediate open forms, the free N-terminal amino group of G4 anchors to the conserved G_352_XMEN_356_ motif through a salt bridge with E_355_ and a hydrogen bond with E_411_ and Q_213_, whereas the carbonyl group of Val-1 forms a hydrogen bond with Y_477_ (Supplementary Table S2 and Fig. 1C). Notably, these residues are known to be fundamental for anchoring the substrate to the active site and positioning it in the correct orientation required for catalysis [31, 33].

The rest of the peptide displayed similar interaction patterns with both the intermediate open and closed CD13 forms, as described in Supplementary Table S2 and Fig. 1C. Notably, in line with the results of the alanine scanning experiment, the side chains of Val-1 and Ala-4 were well inserted into the hydrophobic subsites S1 and S1’, while the side chains of Arg-5 and Arg-6 were involved in electrostatic interactions. Finally, Tyr-7 and Ser-9 were solvent exposed, in the intermediate open CD13 form, and contributed to binding in the closed form (Fig. 1C).

Overall, molecular docking experiments performed on the 3D structures of human CD13 showed that G4 can fit into the catalytic pocket of the enzyme in both closed and intermediate open forms, with the α-amino group anchored to the G_352_XMEN_356_ motif. These data support the hypothesis that the catalytic site is accessible to G4 in the human intermediate open form, and that G4 binding can promote CD13 closure and enzyme activation.

### G4 recognizes soluble forms of CD13, with high affinity and selectivity

**Fig. 2:**
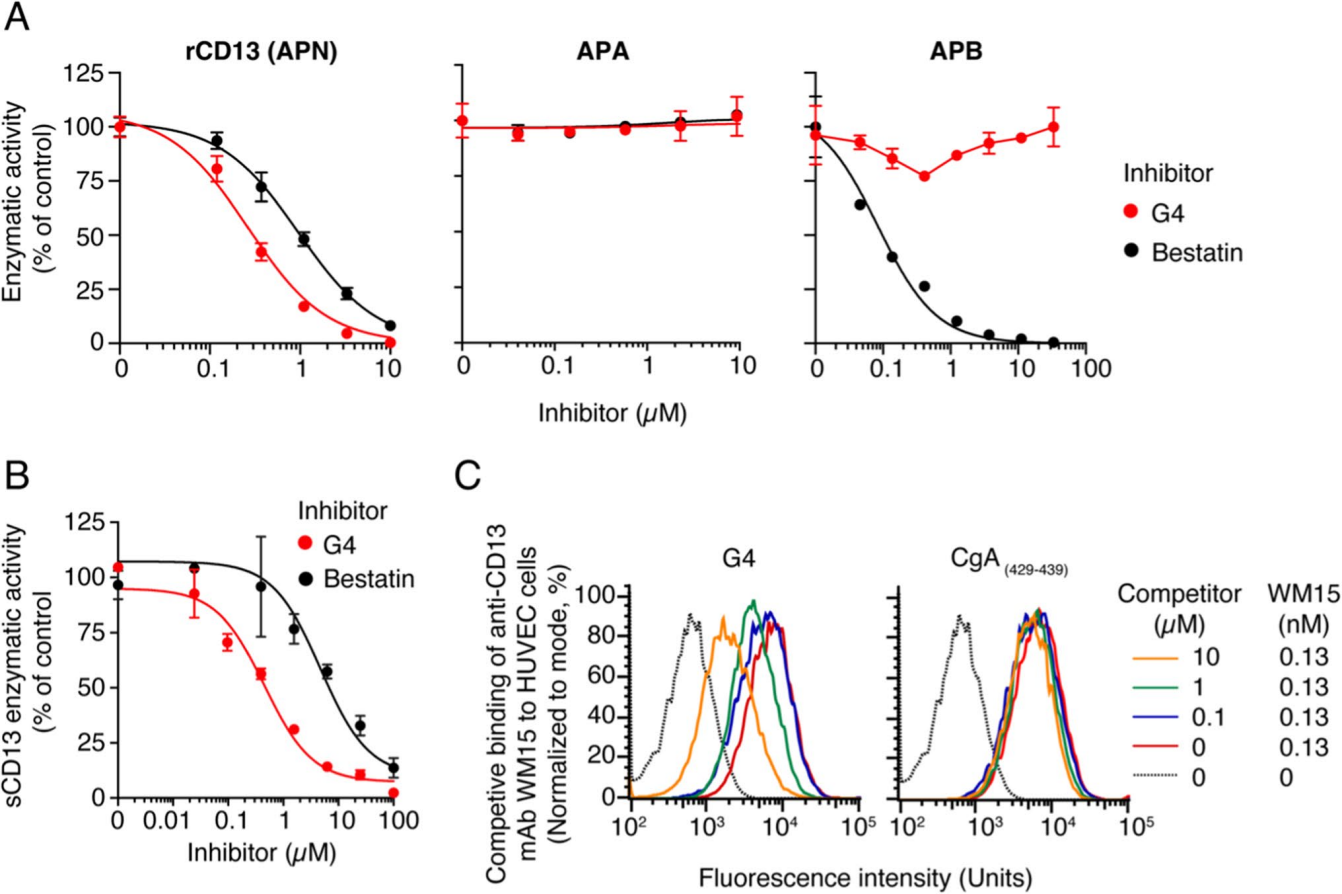
G4 recognizes soluble forms of CD13, with higher affinity and selectivity than bestatin, and the membrane forms expressed on the surface of HUVEC cells. **A** Inhibitory effects of G4 and bestatin on the enzymatic activities of different aminopeptidases (rCD13, APA, and APB). Various amounts of G4 or bestatin were incubated with rCD13 (0.2 µg/mL in 60 mM Tris–HCl pH 7.4), APA (0.2 µg/mL in 60 mM Tris–HCl pH 7.4, 50 mM calcium chloride, 200 mM sodium chloride) or APB (0.2 µg/mL in 60 mM Tris–HCl pH 7.4, 100 mM potassium chloride) and the corresponding substrates at 0.1 mM (l-alanine-7-amido-4-methylcoumarin for rCD13, l-glutamic acid γ-(7-amido-4-methylcoumarin) for APA, and l-arginine-7-amido-4-methylcoumarin for APB). The mixtures were incubated for 30 min at 37 °C, and the cleavage of each substrate was monitored by measuring the fluorescence of methylcoumarin (*λ*_ex_ 341 nm; *λ*_em_ 441 nm) using an Infinite 200 PRO plate reader (Tecan). Three independent experiments were performed for each enzyme. The results of one representative experiment for each enzyme are shown (mean ± SEM, duplicates). **B** Inhibitory effects of G4 and bestatin on soluble CD13 immunocaptured from human serum (sCD13) using microtiter plates coated with anti-CD13 mAb WM15. The microtiter plate was filled with a solution of mAb WM15 (5 μg/ml in DPBS) and incubated overnight at 4 °C. The plate was then washed with DPBS, blocked with 3% bovine serum albumin (BSA) in DPBS, and filled with human serum containing soluble CD13 (diluted 1:2 with DPBS containing 2% BSA). After washing with DPBS, mixtures of G4 or bestatin (0–100 µM in 60 mM Tris–HCl, pH 7.4) and l-alanine p-nitroanilide hydrochloride substrate (4.5 mM in 60 mM Tris–HCl, pH 7.4) were added and left to incubate at 37 °C for 2 h. Substrate cleavage by sCD13 dissociated from mAb WM15 was then monitored by measuring the absorbance at 405 nm in each well using a plate reader (Bio-Rad). The results of one experiment performed in triplicate are shown (mean ± SEM). **C** Effect of G4 on the binding of the anti-CD13 mAb WM15 to HUVEC. HUVEC were seeded at a density of 2 × 10^5^ cells in 100 µL DPBS supplemented with 5% normal human serum (NHS) and incubated with a mixture of G4 peptide (0, 0.1, 1, and 10 µM) and mAb WM15 (0 or 0.13 nM) as indicated. As a negative control, we used the 11-mer peptide CgA_429–439_, which is a human chromogranin A fragment. Cells were incubated for 60 min at 4 °C, washed, and resuspended in 100 µL of DPBS containing 5% NHS and 1 µg/mL FITC-labeled goat anti-mouse IgG secondary antibody. After incubation for 60 min at 4 °C, the cells were washed and analyzed by flow cytometry. Data are expressed as the percentage of counts vs. fluorescence intensity units

To assess the selectivity of G4 for CD13, we then analyzed its inhibitory effects on different soluble aminopeptidases, such as aminopeptidase N (rCD13), aminopeptidase A (APA), and aminopeptidase B (APB). G4 efficiently inhibited rCD13 but not APA and APB (Fig. 2A). In contrast, bestatin, a well-known inhibitor of CD13 [34], inhibited both rCD13 and APB, but not APA. Interestingly, the IC_50_ of G4 for rCD13 was 4–5 fold lower than that of bestatin, indicating that G4 is a more potent and selective inhibitor of rCD13 than bestatin.

To verify that G4 can also interact with natural soluble CD13, we also analyzed its ability to inhibit the enzymatic activity of CD13 immunocaptured from human serum using mAb WM15 (sCD13). As expected, G4 inhibited the activity of natural sCD13 with a potency greater than that of bestatin (Fig. 2B), lending further support to the hypothesis that G4 is an inhibitor of natural soluble CD13, more selective than bestatin.

### G4 recognizes membrane forms of CD13 expressed on the cell surface

To assess whether G4 can also interact with the membrane forms of CD13 expressed by cells, we assessed its ability to compete with the binding of mAb WM15 to HUVEC and HL60 cells, two cell lines that express CD13 on their surface. FACS analysis of antibody binding to HUVEC showed that G4, but not a control peptide (CgA_429-439_), significantly inhibited, albeit partially, the binding of WM15 to HUVEC (Fig. 2C). Similar results were obtained with HL60 cells (data not shown). These findings indicate that G4 recognizes not only the soluble form of CD13 (i.e., the extracellular domain, either natural or recombinant, as shown above), but also the membrane forms of CD13 present on HUVEC and HL60 cell surfaces.

### The G4-TNF fusion protein can interact with CD13 expressed by endothelial cells and with TNF receptors

Considering that CD13, known to be expressed by the tumor vasculature, may represent a target receptor for drug delivery to the tumor vasculature [2, 20], the potential use of G4 as a ligand for drug delivery to tumors was also investigated. To this end, we produced a protein consisting of murine TNF, a potent anticancer cytokine, fused to the C-terminus of G4 using recombinant DNA technology. The fusion protein (called G4-TNF) was a) expressed in *E.coli* cells, b) purified from cell extracts by hydrophobic, ion exchange, and size exclusion chromatography, and c) analyzed by SDS-PAGE, gel-filtration HPLC, mass spectrometry, cytotoxicity assays, and CD13 binding assays. The results of mass spectrometry analysis showed that the final product was characterized by the expected molecular weight (expected, 18,238.21 Da; observed, 18,238.33 Da, as determined by high-resolution mass spectrometry using a Q-Exactive mass spectrometer equipped with a nano-electrospray ion source). SDS-PAGE under reducing conditions showed a single 18 kDa band, as expected for denatured G4-TNF subunits, while the analysis under non-reducing conditions also revealed an additional minor band of 34–36 kDa, likely corresponding to disulfide-bridged dimers (Supplementary Fig. S4A). Gel-filtration HPLC showed a single peak corresponding to that of bioactive trimeric TNF (45–50 kDa), with no evidence of large aggregates or unfolded monomers Supplementary Fig. S4B). The cytotoxic activity of G4-TNF, evaluated using the standard cytolytic assay of L-M fibroblasts in the presence of actinomycin D, was similar to that of TNF (Supplementary Fig. S4C), indicating that the G4 domain of the conjugate did not impair the trimerization of the TNF domain and its interaction of the with TNF receptors. Furthermore, G4-TNF, but not TNF, competed with the binding of mAb WM15 (anti-CD13) to HUVEC to an extent similar to that observed with G4 (Supplementary Fig. S4D). Considering that this competitive assay allows to distinguish the binding of G4-TNF to CD13 from that to TNF-Rs, these results suggest that the TNF moiety of the fusion protein did not impair recognition of CD13 expressed on HUVEC. In conclusion, these results, overall, suggest that G4-TNF can be produced in a trimeric form and that this product is functional in terms of TNF receptor and CD13 recognition in endothelial cells. Thus, this product can be used as a tool to assess whether G4 can recognize CD13 in tumors, which is a prerequisite for its application as a ligand for targeted delivery of drugs to tumors.

### G4-TNF inhibits WEHI-164 fibrosarcoma growth in mice via CD13-dependent mechanism

**Fig. 3:**
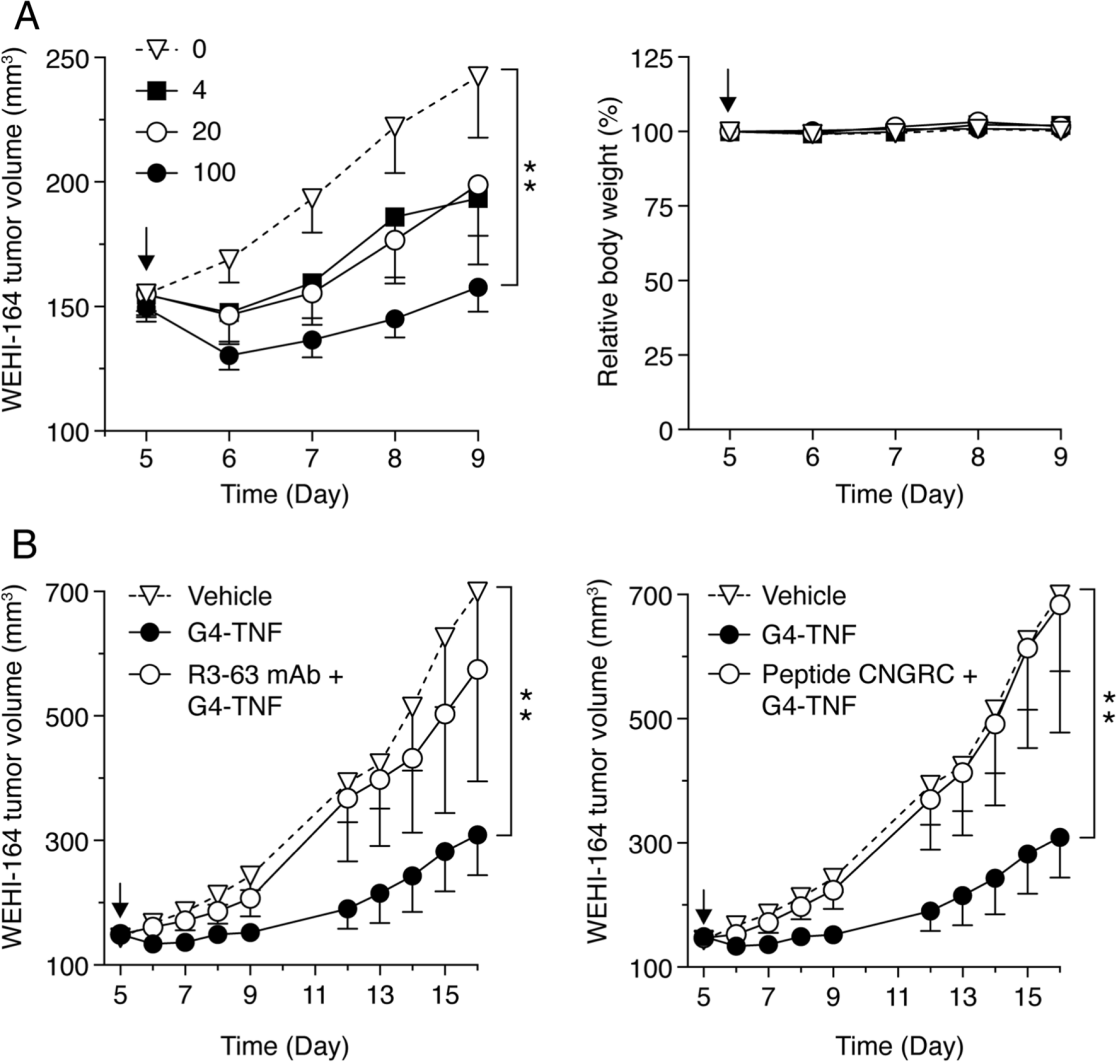
Effect of G4-TNF on tumor growth and body weight of mice bearing WEHI-164 fibrosarcomas. **A** Mice bearing subcutaneous WEHI-164 tumors (5–6 mice/groups) were treated 5 days after tumor implantation with a single intraperitoneal injection of G4-TNF (0, 4, 20, or 100 pg). Tumor volume and body weight after treatment were monitored daily. **B** Effect of mAb R3-63 (anti-murine CD13 antibody, 25 µg) and CNGRC peptide (100 µg) on the antitumor activity of G4-TNF (100 pg) (5–6 mice/group). The arrows indicate the time of treatment. *P < 0.05; **P < 0.01, by Mann–Whitney analysis of the area under the curve for each tumor, with the GraphPad Prism software (mean ± SEM)

To assess whether G4-TNF could recognize CD13 in tumors, we investigated its antitumor activity in mice bearing WEHI-164 fibrosarcomas. The effect of murine TNF (not linked to G4) or TNF linked to CNGRCG (a known CD13 ligand) was also analyzed as negative and positive controls. Preliminary dose-finding experiments, performed with doses of 4, 20, and 100 pg/mouse, showed that a single intraperitoneal injection of 100 pg of G4-TNF was sufficient to induce significant antitumor effects (Fig. 3A). No loss of body weight and no other clinical signs of toxicity (e.g., ruffled fur and abnormal animal behavior) were observed in mice after treatment with G4-TNF. Higher doses were not used to avoid the induction of soluble TNF receptor shedding in circulation. Of note, the delay of tumor growth induced by low-dose G4-TNF was similar to that induced by low doses (50 and 100 pg) of murine CNGRCG-TNF (called NGR-TNF), while a comparable dose (50 pg) of TNF was totally inactive (Supplementary Fig. S5). These results indicate that the fusion of TNF with G4 improves its antitumor activity. To assess whether the improved activity of the fusion protein was mediated by CD13, we co-administered G4-TNF (100 pg) with a molar excess of the neutralizing anti-murine CD13 mAb R3-63 or CNGRC, a peptide known to bind to the active site of CD13 [32, 35]. Both compounds markedly reduced (almost completely) the antitumor activity of G4-TNF (Fig. 3B), suggesting that CD13 was crucially involved in G4-TNF antitumor activity. These results support the hypothesis that G4-TNF can recognize the same CD13 forms recognized by the CNGRC peptide in tumors.

To assess whether endothelial cell killing was a primary mechanism of G4-TNF antitumor activity, we performed cytotoxicity assays with adherent and non-adherent HUVEC cells. No or minimal cytotoxic effects were observed at concentrations lower than 100 pg/ml (i.e., at the pharmacological concentrations obtained in mice injected with 100 pg of G4-TNF) (Supplementary Fig. S6). Thus, although we cannot exclude that some endothelial cells are killed in vivo, it seems unlikely that this represents a primary mechanism of the antitumor activity of G4-TNF. Considering that G4-TNF targets the same receptors of NGR-TNF and that low-dose NGR-TNF is known to enhance endothelial permeability, to induce cytokine-chemokine secretion, and to promote expression of leucocyte adhesion molecules on endothelial cells [36], the antitumor effects triggered by low-dose G4-TNF are likely mediated by similar effects on the tumor vasculature.

### The active site of soluble CD13 is not accessible to G4-TNF

**Fig. 4:**
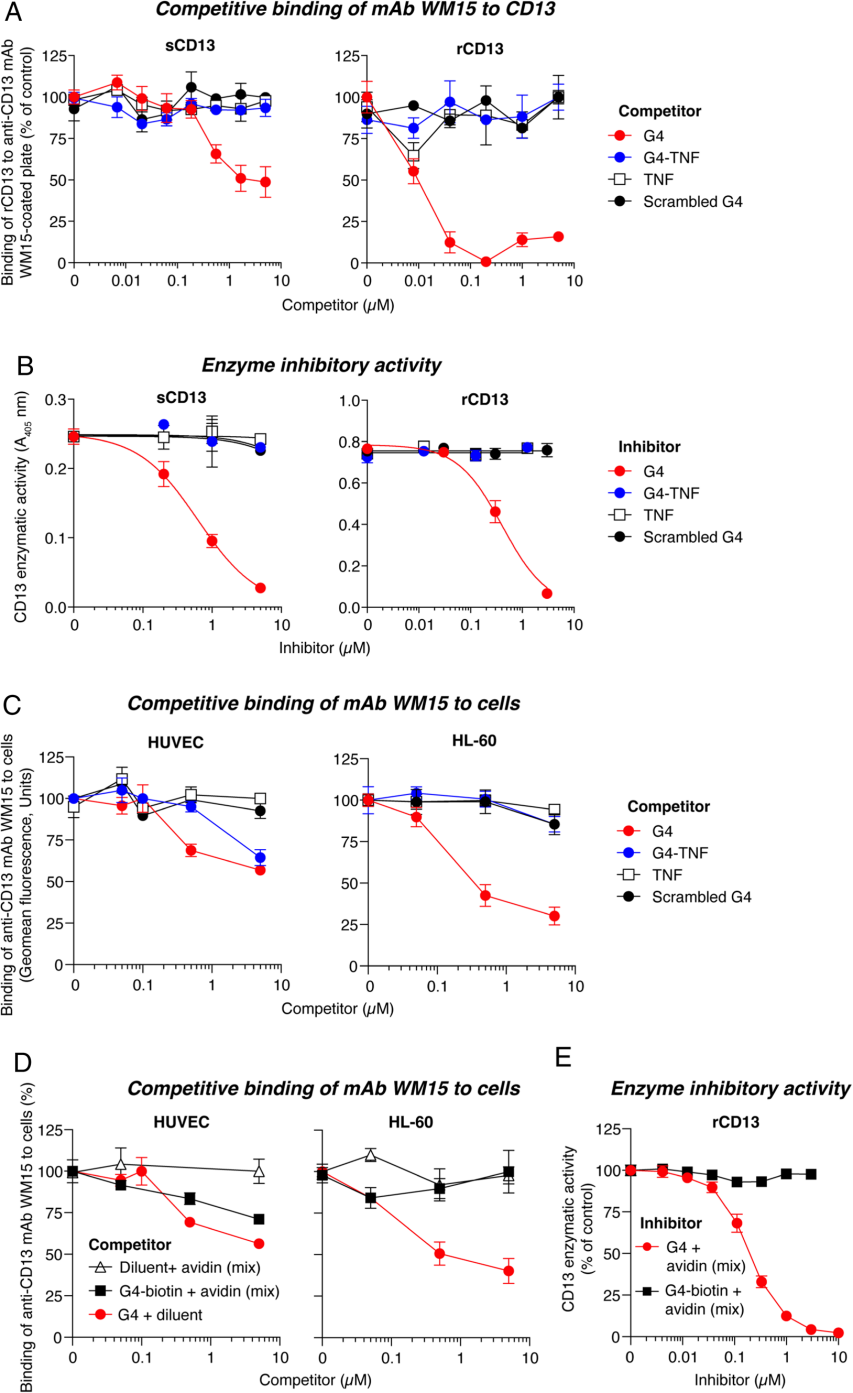
The active site of membrane CD13 expressed by HUVEC, but not that of soluble CD13 or membrane CD13 expressed by HL60 cells, is accessible to G4-TNF. **A** Competitive effects of G4 and G4-TNF on the binding of mAb WM15 (anti-CD13) to sCD13 or rCD13 showing the lack of binding of G4-TNF to natural and recombinant soluble CD13 (sCD13 and rCD13). G4, scrambled G4 (GCRSNCYRVG), and TNF were used as positive (G4) and negative (scrambled G4, TNF) controls. The sCD13 competition assay was performed as follows: microplates were coated with mAb WM15 (5 μg/mL) in DPBS and incubated overnight at 4 °C. Plates were subsequently blocked with 3% BSA in DPBS and filled with a mixture of human serum containing sCD13 (diluted 1:2) and the indicated competitors at different concentrations in DPBS, 0.1% BSA, and 0.05% Tween-20. The mixtures were incubated for 2 h at room temperature and washed with DPBS. To detect bound sCD13, we added an anti-CD13 rabbit polyclonal antibody (1:1000, 1 h at room temperature), washed with DPBS, and added peroxidase-labeled goat anti-rabbit secondary antibody (1:2000, 1 h). Bound peroxidase was detected using o-phenylendiamine chromogenic substrate. The absorbance of o-phenylenediamine was measured at 490 nm using an ELISA plate reader (Bio-Rad). Cumulative results of two independent experiments, each performed in duplicate, are shown (mean ± SEM). The rCD13 competition assay was performed as described in Supplementary Fig. S2 (one experiment in duplicate, mean ± SEM). **B** Inhibitory effects of G4 or G4-TNF on the sCD13 or rCD13 enzymatic activity (one experiment performed in duplicates; mean ± SEM). **C** Competition of mAb WM15 binding to HUVEC or HL-60 cells with G4 and G4-TNF. Scrambled G4 and TNF were used as negative controls. Cumulative results of three (HUVEC cells) or two (HL-60 cells) independent experiments, each performed in duplicate or triplicate (mean ± SEM). **D** Competitive binding of mAb WM15 (anti-CD13) to HUVEC and HL-60 cells with G4 and G4-Avidin complex prepared by mixing a G4-biotin conjugate with avidin (see “Methods). Avidin was also used as control. The experiment was performed in duplicate (mean ± SEM). **E** Inhibitory effects of G4 and G4-Avidin on rCD13 enzymatic activity. The assay was performed as described in Supplementary Fig. S2. The results of one experiment, performed in duplicate, are shown (mean ± SEM)

To clarify how an extremely low dose of G4-TNF can target CD13 in tumors despite the presence of large amounts of CD13 in the blood and in normal tissues, we first examined the interaction of G4-TNF with soluble CD13 extracted from human serum (sCD13). Interestingly, although G4 competed for the binding of WM15 to sCD13, no competition was observed with G4-TNF (Fig. 4A, left). Similar results were obtained with recombinant soluble CD13 (rCD13) (Fig. 4A, right), suggesting that the G4-binding site in the soluble form of CD13, either natural or recombinant, is accessible to G4 but not to G4-TNF. Accordingly, G4, but not G4-TNF, inhibited the enzymatic activity of both natural and recombinant soluble CD13 (Fig. 4B), further supporting the hypothesis that their catalytic site is accessible to G4 but not to G4-TNF. These results may explain why the circulating soluble CD13 did not impair the interaction of G4-TNF with CD13 expressed in tumors in the in vivo experiments described above.

### The active site of CD13 expressed by HUVEC, but not that expressed by HL60 cells, is accessible to G4-TNF

Considering the above results, we then investigated the binding of G4-TNF to CD13 expressed by endothelial cells and other cells, such as human leukemic HL-60 cells (a CD13-positive “non-vascular” cell line). To this aim, we compared the capability of G4-TNF to compete the binding of mAb WM15 to these cells. Interestingly, G4-TNF competed the binding of mAb WM15 to endothelial cells but not to HL-60 cells (Fig. 4C), suggesting that only the CD13 form expressed by HUVEC has a site accessible to G4-TNF.

### The active site of CD13 expressed by HUVEC, but not that expressed by HL60 cells, is accessible to G4-avidin complex

To assess whether this phenomenon is a peculiarity of G4-TNF or if other G4-protein conjugates behave in the same manner, we performed a similar experiment on HUVEC and HL-60 cells, using a G4-avidin complex instead of G4-TNF (prepared by mixing a G4-biotin conjugate with avidin). Interestingly, also this complex, competed the binding of mAb WM15 to endothelial cells, but not to HL-60 cells (Fig. 4D). Furthermore, this complex, like G4-TNF, could not inhibit the enzymatic activity of recombinant soluble CD13 (Fig. 4E). It appears, therefore, that the binding site of CD13 molecules expressed by endothelial cells, but not that expressed by HL-60 cells, is accessible to large G4-protein conjugates and complexes.

### Endothelial cells express high levels of a catalytically inactive form of CD13 that is selectively recognized by G4-TNF

**Fig. 5:**
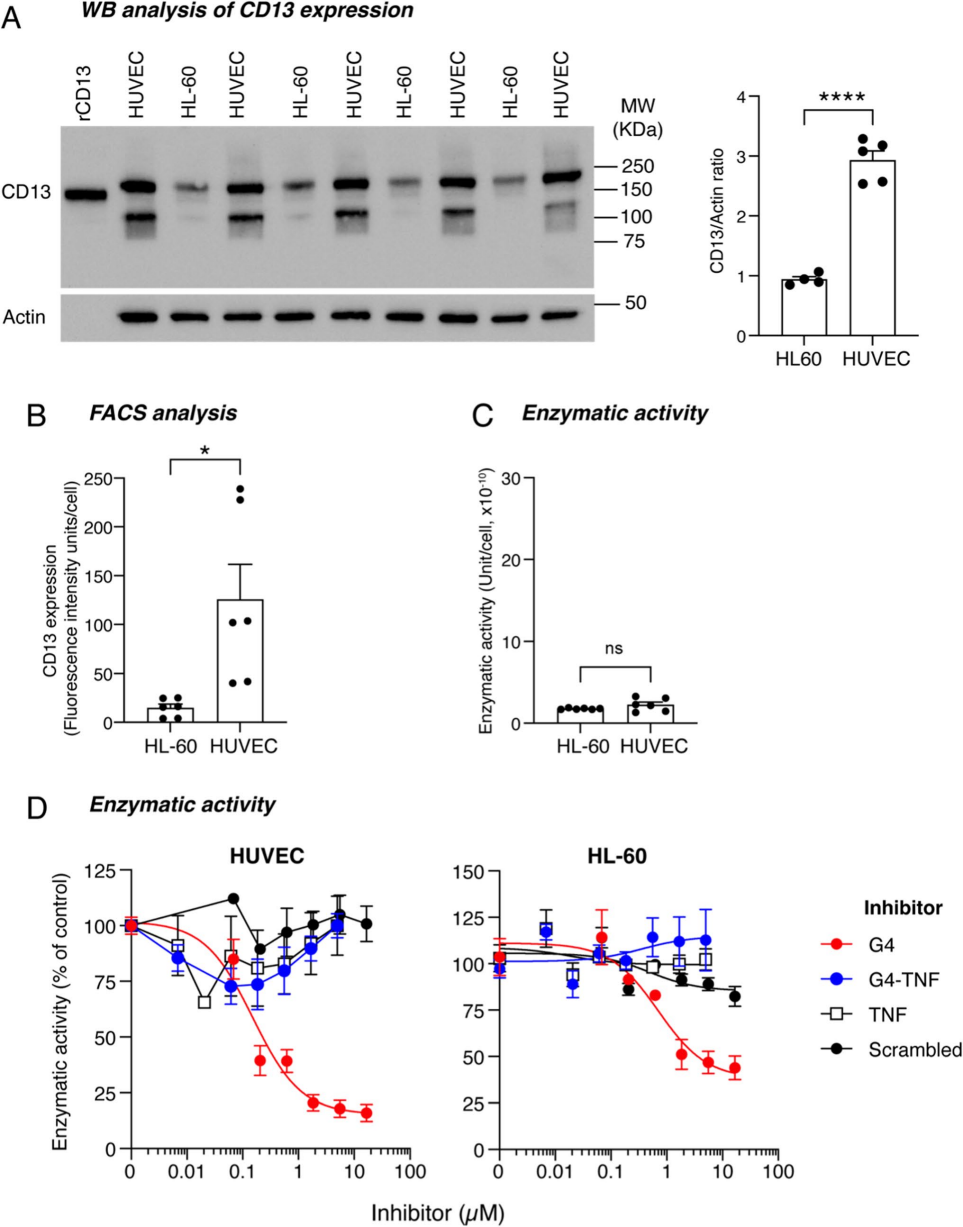
Quantification of CD13 expressed by HL-60 and HUVEC by western blotting and FACS analyses, and determination of their enzymatic activity. **A** Western blotting (WB) analysis of CD13 expression in HL-60 cells and HUVEC. For both cell lines, 2 µg of cell lysates were loaded in each well (*n* = 4–5 replicates, as indicated). After blotting, the membrane was cut and analyzed for CD13 and actin expression as described in Material and methods. The results of one representative experiment, out of three independent experiments performed, are shown (bars, ratio of CD13/actin band intensity, mean ± SEM). Recombinant human CD13 (rCD13) (20 ng) was loaded as positive control. MW, molecular weight markers. **B** FACS analysis of CD13 expression in HUVEC and HL-60 cells. The cells were incubated with mAb WM15 (10 μg/mL) and stained with a FITC-labeled goat anti-mouse IgG (H + L) secondary antibody. Data were expressed as fluorescence intensity and normalized to the number of cells. The cumulative results of three independent experiments are shown (each performed in duplicate; mean ± SEM). **C** Enzyme activity assay of CD13 expressed in HUVEC and HL-60 cells. Cells were seeded into 96-well microtiter plates (3 × 10^5^ cells/well) and incubated with l-alanine p-nitroanilide hydrochloride substrate (0.5 mM in DPBS) for 30 min at 37 °C. Absorbance at 405 nm was measured using a plate reader. The enzyme units in each sample were calculated using rCD13 as a standard and were normalized to the number of cells. Cumulative results of three independent experiments, each performed in duplicate, are shown (mean ± SEM). **D** Inhibition of the enzymatic activity of CD13 expressed in HUVEC and HL-60 cells by G4 and G4-TNF. Cells were seeded into 96-well microtiter plates (3 × 10^5^/well) and incubated with the l-alanine p-nitroanilide hydrochloride substrate and different amounts of G4-TNF, G4, TNF, or scrambled G4 peptide (30 min at 37 °C). The absorbance was measured at 405 nm using a plate reader. The results of one experiment performed in duplicate are shown (mean ± SEM)

To gain insight into the structural and functional properties of CD13 recognized by G4-TNF and G4-avidin on endothelial cells, we compared the enzymatic activity of CD13 expressed by HUVEC with that expressed by HL60 cells. First, we quantified the relative amount of CD13 expressed by these cells by western blot analysis of cell lysates with mAb WM15, as well as FACS analysis with the same antibody. The results showed that HUVEC expressed a larger amount of CD13 on their surface than HL60 cells (Fig. 5A and B). Then we determined cell-associated enzymatic activity by incubating each cell line with l-alanine-p-nitroanilide, a chromogenic substrate of CD13. This assay showed that despite HUVEC and HL60 cells express markedly different amounts of CD13 antigen, their catalytic efficiency was similar (Fig. 5C), suggesting that a large fraction of CD13 molecules present on HUVEC were catalytically inactive. Interestingly, although G4-TNF partially inhibited the binding of mAb WM15 to HUVEC, as shown above (see Fig. 5C), this protein did not inhibit the CD13 enzymatic activity associated with either cell line (Fig. 5D). These observations suggest that G4-TNF recognizes only the catalytically inactive form of CD13 expressed by HUVEC and not the catalytically active form expressed by both cell lines.

**Fig. 6:**
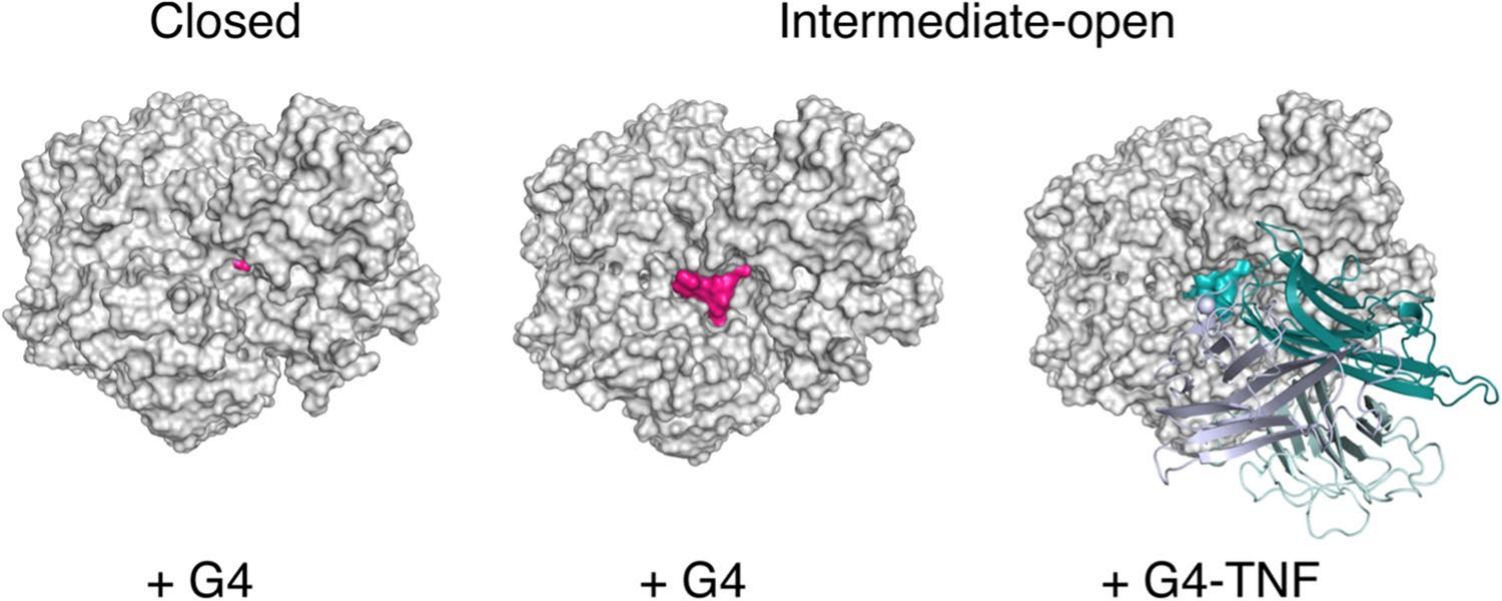
Representative model of the interaction of G4 and G4-TNF with different human CD13 forms. Docking pose of G4 bound to CD13 in a closed and intermediate open form (left and center); model of G4-TNF in complex with the intermediate open form of CD13 (right). G4 and CD13 are represented by the pink and white surfaces, respectively. G4-TNF is shown in cyan

We hypothesized that this phenomenon depends on a differential accessibility of the binding pocket associated with the different conformations of CD13. A computational model showed that while G4 can fit into the pocket of open CD13 as well as in the narrow cavity of the closed CD13 forms (see Fig. 1C), G4-TNF could only accommodate into an open form because of steric hindrance caused by the TNF domain (Fig. 6). This model provides additional support for the hypothesis that the catalytically inactive form recognized by G4-TNF in endothelial cells adopts an open conformation.

### Microtiter plates coated with G4-avidin or G4-streptavidin complexes promote endothelial cell adhesion

**Fig. 7:**
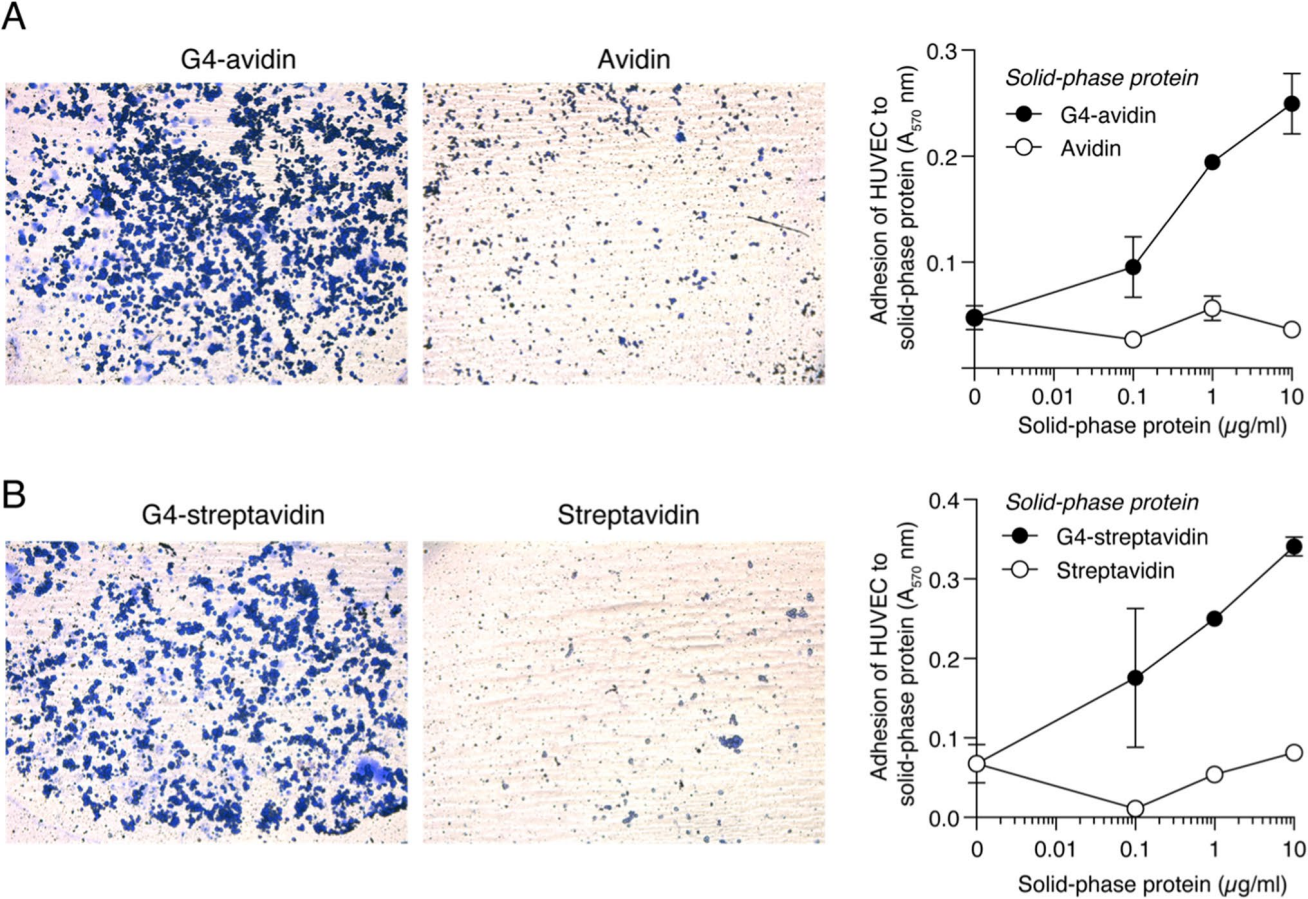
Adhesion of HUVEC cells to microtiter plates coated with G4-avidin or G4-streptavidin conjugates. Representative images of wells coated with 10 µg/mL of G4-avidin (**A**) or G4-streptavidin (**B**) complexes. Wells coated with avidin or streptavidin were used as negative controls. The absorbance of crystal violet-stained adherent cells is also shown. Two independent experiments, each performed in duplicate, were performed. Images were taken at the center of a 96-well microtiter plate using a 10 × objective. The results of one representative experiment are shown (mean ± SEM)

Finally, to assess whether the catalytically inactive form of CD13 has a biological function, we tested the adhesion of HUVEC to microtiter plates coated with G4-avidin or G4-streptavidin complexes. The results showed that both complexes, but not avidin or streptavidin, promoted endothelial cell adhesion (Fig. 7), suggesting that the engagement of the catalytically inactive form by the G4 peptide can affect endothelial cell biology.

## Discussion

In the present study, we identified and optimized a novel inhibitor of CD13 (G4), consisting of a disulfide-bridged 9-mer peptide with the VGCARRYCS sequence. The results of steady-state kinetic analysis of the recombinant CD13 ectodomain and the Lineweaver–Burk analysis of enzyme inhibition data are consistent with a competitive mechanism of inhibition (*K*_*i*_ ~ 22 nM), suggesting that G4 interacts with the catalytic site of the enzyme. The affinity of G4 for CD13 is 4–5 fold higher than that of bestatin, a well known and widely investigated inhibitor of CD13 [34]. Furthermore, G4 efficiently inhibited recombinant CD13, but not recombinant aminopeptidase A (APA) or aminopeptidase B (APB), whereas bestatin inhibited both CD13 and APB, indicating that G4 is more selective for CD13 than bestatin.

Acetylation of the α-amino group of G4 completely abrogated its inhibitory activity, indicating a crucial role of the N-terminal region of the peptide in enzyme recognition. Furthermore, substitution of Val-1, Arg-5, and Arg-6 with Ala markedly reduced its inhibitory activity, suggesting that these amino acid residues play important roles in the G4/CD13 interaction. Accordingly, a molecular docking model suggested that Val-1 interacts with Glu-355, Glu-411, and Gln-213 of human CD13, and that Val-1 and Ala-3 fit into the S1 and S1’ subpockets of CD13, while Arg-5 and Arg-6 form a network of electrostatic interactions within the catalytic pocket.

Considering that the CNGRC peptide is a CD13 ligand widely used by many investigators for targeting drugs to tumors [21], the fact that G4, like other NGR-containing peptides, binds to the catalytic pocket of CD13 [32] suggests that also G4 might be used as a ligand for delivering drugs to tumors. This view is supported by the results of in vivo studies showing that G4 fused to TNF, an antitumor cytokine, can enhance the therapeutic effects of this cytokine in the WEHI-fibrosarcoma murine model. Notably, in this experimental model, systemic administration of an extremely low dose of the G4-TNF fusion protein (100 pg) was sufficient to induce antitumor effects, as previously observed with NGR-TNF and its SNGR-TNF-derivative [24, 37]. The role of CD13 as a targeting receptor in this experimental setting is supported by the observation that the antitumor activity of G4-TNF was completely abrogated by an anti-CD13 antibody or by an excess of CNGRC peptide. Considering that a comparable dose of TNF is inactive in the WEHI-fibrosarcoma model [38], these results suggest that the G4 moiety of the conjugate, like CNGRC, can indeed enable the targeted delivery of TNF to the tumor vasculature.

The question arises as to whether G4-TNF can specifically recognize the endothelial form of CD13 in tumor vessels or whether it can also bind other forms of CD13 in the body. The results of binding studies and enzyme inhibition assays performed with the free G4 peptide showed that this compound can recognize various forms of CD13, including soluble plasma CD13, membrane CD13 expressed by endothelial cells, and membrane CD13 expressed by HL60 promyelocytic leukemia cells. These findings argue, apparently, against the hypothesis of a specific mechanism. However, the results of binding studies performed with G4-TNF showed that this conjugate, in contrast to free G4, can recognize only the CD13 form expressed by endothelial cells, and not the soluble CD13 isolated from plasma, or the membrane CD13 expressed by HL60 cells, indicating a specific mechanism.

The distinct behavior observed between free G4 and G4-TNF in the recognition of soluble CD13 can be rationalized using our computational models. They showed that G4 could readily fit into the narrow binding pocket of the closed/active form of soluble CD13. In contrast, G4-TNF is unable to accommodate the closed form of the enzyme, likely because of steric hindrance caused by the TNF domain. However, while the TNF domain seems to hinder the access of G4-TNF to the binding pocket of soluble CD13, it does not hinder the binding of the conjugate to CD13 expressed by endothelial cells, suggesting that in this case, the binding site has a conformation with a higher accessibility. The selective recognition of the endothelial form of CD13, which is known to be upregulated in tumors, is not limited to G4-TNF, as other G4-protein conjugates, such as G4-biotin/streptavidin and G4-biotin/avidin complexes, could selectively recognize this CD13 form and not that expressed by the HL-60 cells in our in vitro assays.

These findings raise another important question regarding the catalytic activity and biological function of the endothelial form recognized by G4-TNF or G4-avidin. The results of biochemical studies performed to address this question show that although endothelial cells (HUVEC) express a > tenfold larger amount of CD13 on their surface compared to HL60 cells, their catalytic efficiencies are similar. This observation suggests that a large fraction of CD13 molecules on the endothelial cell surface are catalytically inactive. This view is also supported by the observation that G4-TNF can inhibit the binding of the anti-CD13 monoclonal antibody WM15 to HUVEC but not the CD13 enzymatic activity associated with these cells. In other words, endothelial cells express a large amount of catalytically inactive CD13 (recognized by WM15 and G4-TNF) and a low amount of catalytically active CD13 (recognized by WM15, but not by G4-TNF).

It has been previously shown that CD13 is a dimeric glycoprotein with an arch-like structure and monomers that can assume closed or open conformations [8]. Furthermore, crystal structure analyses of the dimeric-ectodomain of human CD13 in the presence of angiotensin IV or peptidomimetic inhibitors (bestatin or amastatin) have suggested that rapid interconversion between the open and closed forms of the ectodomains is involved in both proteolytic and signal transduction mechanisms [8]. Therefore, one possibility is that the form recognized by G4-TNF on endothelial cells corresponds to a protein that is in a constitutively open conformation. The structural determinants of this molecular conformation in endothelial cells are unknown. It is possible that in these cells, CD13 undergoes differential post-translational modifications (e.g., phosphorylation or glycosylation) [39, 40] or forms complexes with a membrane-linked allosteric inhibitor capable of stabilizing a catalytically inactive-accessible form.

Considering that CD13 is upregulated in the tumor vasculature, our findings raise another important question regarding the potential role of the catalytically inactive form of CD13 expressed by endothelial cells in tumor vascular biology. Previous studies have shown that anti-CD13 antibodies impair angiogenesis in animal models, and that CD13-null mice have reduced angiogenic responses to growth factors [41], suggesting that CD13 plays an important role in angiogenesis. Other investigators have shown that CD13 expressed on endothelial cells and monocytes can mediate homotypic cell adhesion in a manner independent on enzymatic activity [12]. Another study has shown that the migration capacity of human lung adenocarcinoma cells expressing a genetically modified, enzymatically inactive form of CD13 is not fully impaired, suggesting that CD13 can enhance cancer cell motility by both enzyme-dependent and enzyme-independent mechanisms [42]. Interestingly, our results showed that microtiter plates coated with G4-biotin/avidin or streptavidin conjugates can promote endothelial cell adhesion, suggesting that the catalytically inactive form can contribute to the regulation of endothelial cell biology. Therefore, it seems that the functional role of CD13 is not exclusively dependent on its catalytic activity and that both enzymatically active and inactive forms of CD13 expressed by endothelial cells may have a functional role. Further studies are necessary to assess this possibility.

In conclusion, we have identified a novel peptide (G4) that can bind and block the active site of soluble and membrane forms of CD13 with high affinity and selectivity, but not after coupling to proteins such as TNF, avidin, or streptavidin, likely due to steric hindrance mechanisms. Furthermore, we have shown that endothelial cells express a catalytically inactive form of CD13, characterized by a binding site that is accessible to G4-protein conjugates.

The free G4 peptide may represent a novel inhibitor of catalytically active CD13 that is more efficient and selective than bestatin. G4 may also represent, upon conjugation with proteins, an efficient and selective ligand of the catalytically inactive form of CD13 expressed by endothelial cells, potentially useful for delivering drugs to tumors.

Further studies are necessary to elucidate the structure and function of the enzymatically inactive CD13. Furthermore, studies with an appropriate peptide–protein conjugate capable of selectively recognizing the catalytically inactive form are necessary to assess the presence or absence of this protein in other organs in the body. The G4-protein conjugates described herein may represent useful tools and probes for studies aimed at investigating the structural determinants and functional properties of the catalytically inactive form of CD13 expressed by endothelial cells in tumors.

### Supplementary Information

Below is the link to the electronic supplementary material.

Supplementary file1 (DOCX 1654 KB)

## Data Availability

All data generated or analyzed during this study are included in this published article and its supplementary information files. The raw data are available upon request from the corresponding author.
