# Supplementary material for: A novel aminopeptidase N/CD13 inhibitor selectively targets an endothelial form of CD13 after coupling to proteins
Source: Cell Mol Life Sci. 2024 Jan 30;81(1):68. doi: 10.1007/s00018-023-05102-1 (PMC10827914; doi:10.1007/s00018-023-05102-1)
Supplement: Supplementary file 1 — Supplementary file1 (DOCX 1654 KB) [file 18_2023_5102_MOESM1_ESM.docx]

**A novel aminopeptidase N/CD13 inhibitor selectively targets an endothelial form of CD13 after coupling to proteins**

Giulia Anderluzzi, Michela Ghitti, Anna Maria Gasparri, Giulia Taiè, Angelina Sacchi, Alessandro Gori, Annapaola Andolfo, Federica Pozzi, Giovanna Musco, Flavio Curnis and Angelo Corti

**Supplementary Materials and Methods**

**Peptide synthesis, purification, and characterization**

The peptides were synthesized, cyclized, purified and analyzed by ProteoGenix or in-house (see **Supplementary Table S1**). In house production of peptides used in the alanine scanning experiment of VGCGRRYCSN, the VGCGRRYCS peptide used for CD13 digestion experiments (Lot#B), and the negative control peptide VAHQLQALRRG (CgA_429-439_) were synthesized by stepwise microwave-assisted Fmoc-solid-phase method on a Biotage® Initiator+ Alstra™ peptide synthesizer, operating in a 0.12 mmol scale on a pre-loaded 2-chlorotrityl chloride resin (0.6 mmol/g). The resin was swelled before use with an NMP/DCM mixture. Activation and coupling of Fmoc-protected amino acids were performed using Oxyma 0.5 M in DMF/0.5 M DIC in DMF (1:1:1), with a 5-equivalent excess over the initial resin loading. Coupling steps were performed for 10 min at 50°C. Deprotection steps were performed by treatment with a 20% piperidine solution in DMF at room temperature. Following each coupling or deprotection step, the peptidyl resin was washed 4 times with 5 ml of DMF. The final peptide was cleaved from the resin using a mixture containing 90% TFA, 5% water, 2.5% thioanisole, and 2.5% TIS (3 h at room temperature). After precipitation in cold diethyl ether, crude peptides were centrifuged and washed with cold diethyl ether to remove scavengers. Peptides was then dissolved in 50% water/acetonitrile containing 0.07% TFA and purified by preparative reverse-phase high performance liquid chromatography (RP-HPLC) using a Phenomenex Jupiter C18 90Å (5 µm, 250 x 21.2 mm) column connected to a Shimadzu Prominence HPLC system, equipped with a multichannel detector. The column was eluted with mobile phase A (3% acetonitrile, 0.07% TFA in water) and mobile phase B (70% acetonitrile, 0.07% TFA in water) using the following chromatographic method: 0% B (7 min), 0−30% B linear-gradient (40 min); flow rate, 14 ml/min. The chromatographic separation was monitored in the 220-320 nm range and processed with the LabSolutions software. RP-HPLC fractions were pooled and lyophilized. The resulting product, corresponding to the linear peptide, was dissolved in sodium phosphate buffer, pH 7.4, (0.5 mM, final concentration) and mixed with a 10 mM hydrogen peroxide solution (10 μM, final concentration) to favor disulfide-mediated cyclization. The mixture was left to incubate under stirring until the complete conversion of linear precursor into the cyclic peptide occurred (as verified by analytical RP-HPLC). The pH of the solution was then adjusted to pH 3 using a 10% TFA solution, and the purification process was carried out once again by RP-HPLC. The resulting lyophilized peptides were dissolved in sterile water, aliquoted, and stored at -25°C. The identity of each peptide was confirmed by electrospray ionization mass spectrometry analysis using an LTQ-XL Orbitrap spectrometer (Thermo Scientiﬁc) (see **Supplementary Table S1**). Peptide purity was assessed by analytical RP-HPLC using a Phenomenex Jupiter C18 90Å (5 µm, 150 x 4.6 mm) column essentially as described above.

**TNF cytolytic assay based on L-M cells**

The cytolytic assay used for testing the bioactivity of G4-TNF and TNF was based on the L-M murine fibroblast cell line (clone CCL-1.2; ATCC). L-M cells were cultured in DMEM medium supplemented with 2 mM glutamine, 100 U/mL penicillin, 100 µg/mL streptomycin, 0.25 µg/mL amphotericin-B and 10% fetal bovine serum. The cells were detached with a trypsin-EDTA solution and plated in 96-well flat-bottom plate for cell culture (30000 cells/well, 100 µL/well) in complete medium and incubated overnight at 37°C, 5% CO_2_. Fifty µL of actinomycin-D (2 µg/mL, final concentration) and 50 µL of G4-TNF or TNF solutions in complete medium, were then added to each well and left to incubate for 24 h at 37°C, 5% CO_2_. Twenty µL aliquots of PrestoBlue Cell Viability Reagent, were then added to the wells and left to incubate for 1 h at 37°C, 5% CO_2_. The absorbance at 570 nm and 595 nm was then read using a microtiter plate reader; the A_570_-A_595_ was then calculated and plotted versus TNF or G4-TNF concentration.

**Supplementary Table S1. Electrospray ionization mass spectrometry analysis (ESI-MS) and RP-HPLC analysis of the peptides used in this work.**

| **Peptide** | **Produced**  **by** | **Monoisotopic mass**  **by ESI-MS (Da)** | | **Peptide purity by HPLC (%)** |
| --- | --- | --- | --- | --- |
|  |  | ***Expected*** | ***Found*** |  |
|  |  |  |  |  |
| VGCGRRYCSN | Proteogenix | 1111.46 | 1111.47 ^a)^ | 95.3 ^b)^ |
|  |  |  |  |  |
| **A**GCGRRYCSN | In-house | 1083.43 | 1083.43 ^c)^ | 96.0 ^d)^ |
| V**A**CGRRYCSN | In-house | 1125.48 | 1125.48 ^c)^ | 95.2 ^d)^ |
| VG**A**GRRY**A**SN | In-house | 1049.53 | 1049.53 ^c)^ | 96.1 ^d)^ |
| VGC**A**RRYCSN | In-house | 1125.43 | 1125.48 ^c)^ | 93.1 ^d)^ |
| VGCG**A**RYCSN | In-house | 1026.40 | 1026.41 ^c)^ | 96.4 ^d)^ |
| VGCGR**A**YCSN | In-house | 1026.40 | 1026.40 ^c)^ | 95.4 ^d)^ |
| VGCGRR**A**CSN | In-house | 1019.43 | 1019.44 ^c)^ | 95.1 ^d)^ |
| VGCGRRYC**A**N | In-house | 1095.47 | 1095.47 ^c)^ | 95.9 ^d)^ |
|  |  |  |  |  |
|  |  |  |  |  |
| VGCARRYCSN | Proteogenix | 1125.43 | 1125.35 ^a)^ | 96.3 ^b)^ |
| ac-VGCARRYCSN | Proteogenix | 1167.49 | 1167.45 ^a)^ | 95.5 ^b)^ |
| VGCARRYCS (G4, Lot #A)^e)^ | Proteogenix | 1011.43 | 1011.50 ^a)^ | 99.4 ^b)^ |
| VGCARRYCSGGSGGGSGK-bio | Proteogenix | 1882.80 | 1882.85 ^a)^ | 95.7 ^b)^ |
| VGCARRYC | Proteogenix | 924.40 | 924.60 ^a)^ | 93.8 ^b)^ |
| GCARRYCSN | Proteogenix | 1026.41 | 1026.60 ^a)^ | 93.8 ^b)^ |
| GCARRYCS | Proteogenix | 912.36 | 912.50 ^a)^ | 93.1 ^b)^ |
| CARRYC | Proteogenix | 768.31 | 768.50 ^a)^ | 91.2 ^b)^ |
|  |  |  |  |  |
|  |  |  |  |  |
| GCRSNCYRVG | Proteogenix | 1111.46 | 1111.45 ^a)^ | 98.1 ^b)^ |
| VGCARRYCS (G4, Lot #B)^f)^ | In-house | 1011.43 | 1011.50 ^c)^ | 91.9 ^d)^ |
| VAHQLQALRRG (CgA_429-439_) | In-house | 1247.72 | 1247.20 ^c)^ | 95.9 ^d)^ |
| CSGIGSGGC | In-house | 737.25 | 737.25 ^c)^ | 94.1 ^d)^ |
| CNGRC | In-house | 549.17 | 549.18 ^c)^ | 95.1 ^d)^ |
|  |  |  |  |  |

1. Mass spectrometry analysis performed by Proteogenix.
2. RP-HPLC analysis performed by Proteogenix using an Agela 100-5C18 column (4.6 x 250 mm, 5 µm).
3. Mass spectrometry analysis performed in-house using a Q-Exactive mass spectrometer (Thermo Scientific, Bremen, Germany). The mass value reported for each Ala-scan peptide corresponds to the major component in the mass spectrum; an additional minor component of 912.37 Da, corresponding to peptides with valine deletion, was also observed in peptides analyzed in-house (see for example the *Panel T=0 min of* **Supplementary Fig. S3**).
4. Analyzed in-house using a Shimadzu Prominence HPLC system, connected to a Shimadzu Shimpack GWS 5 μm C18 90Å column (150 x 4.6 mm) connected to a diode array detector.
5. Lot #A: used in most of the in vitro experiments reported in the manuscript.
6. Lot #B: used in the experiment reported in **Supplementary Fig. S3**

**Supplementary Table S2. Analysis of the electrostatic interactions of the docking poses**^(a)^

| **VGCARRYCS** | |  | **CD13_closed_** |  | **CD13_intermediate open_** |
| --- | --- | --- | --- | --- | --- |
| Res 1 | Val-1_Hα_ |  | E355_OD_ |  | E355_OD_ |
|  |  |  | E411_OD_ |  | E411_OD_ |
|  |  |  | Q213_OE_ |  | Q213_OE_ |
|  | Val-1_O_ |  | Y477_HG_ |  | Y477_HG_ |
| Res 2 | Gly-2_H_ |  | A353_O_ |  | A353_O_ |
|  | Gly-2_O_ |  | R381_HH_ |  |  |
| Res 3 | Cys-3_O_ |  |  |  | R381_HH_ |
| Res 4 |  |  |  |  |  |
| Res 5 | Arg-5_O_ |  | N438_HE_ |  | R442_HH_ |
|  | Arg-5_HH_ |  | E380_OD_ |  |  |
|  |  |  | E418_OD_ |  | E418_OD_ |
|  |  |  | T384_OG_ |  |  |
| Res 6 | Arg-6_HH_ |  | E380_OD_ |  | F472_O_ |
|  | Arg-6_O_ |  |  |  | R442_HH_ |
| Res 7 | Tyr-7_O_ |  | N900_HE_ |  | R381_HH_ |
|  | Tyr-7_OH_ |  | S899_OG_ |  |  |
| Res 8 | Cys-8_O_ |  | R363_HH_ |  |  |
| Res 9 | Ser-9_OH_ |  | N900_HE_ |  | N900_HE_ |
|  | Ser-9_O_ |  | R363_HH_ |  |  |
|  |  |  | S861_HG_ |  |  |

a) Interactions engaged by VGCARRYCS with the closed and intermediate open form of CD13 are reported. H and O indicate hydrogen and oxygen atom of peptide bond; OE, OG, and OD denote amide, hydroxyl and carboxylate oxygen, respectively; Hα, HE, HH, and HG indicate a-amino group, amide, guanidinium, and hydroxyl proton, respectively.

A


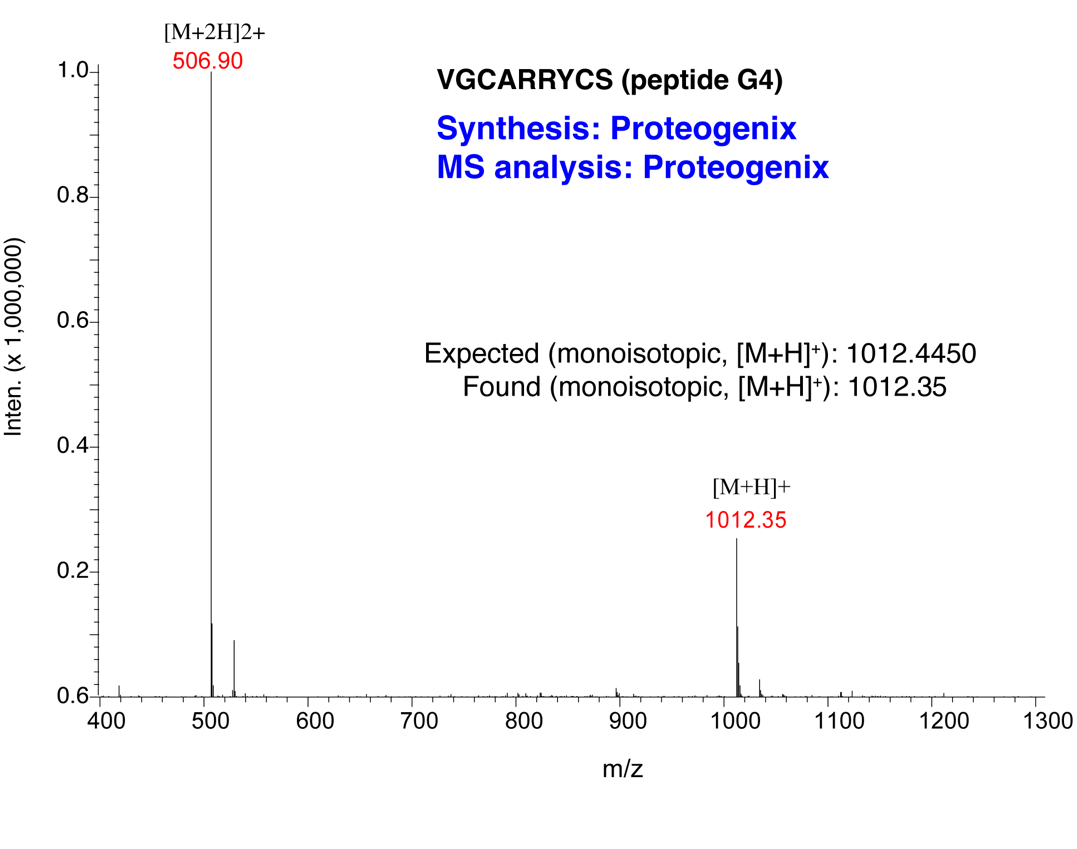

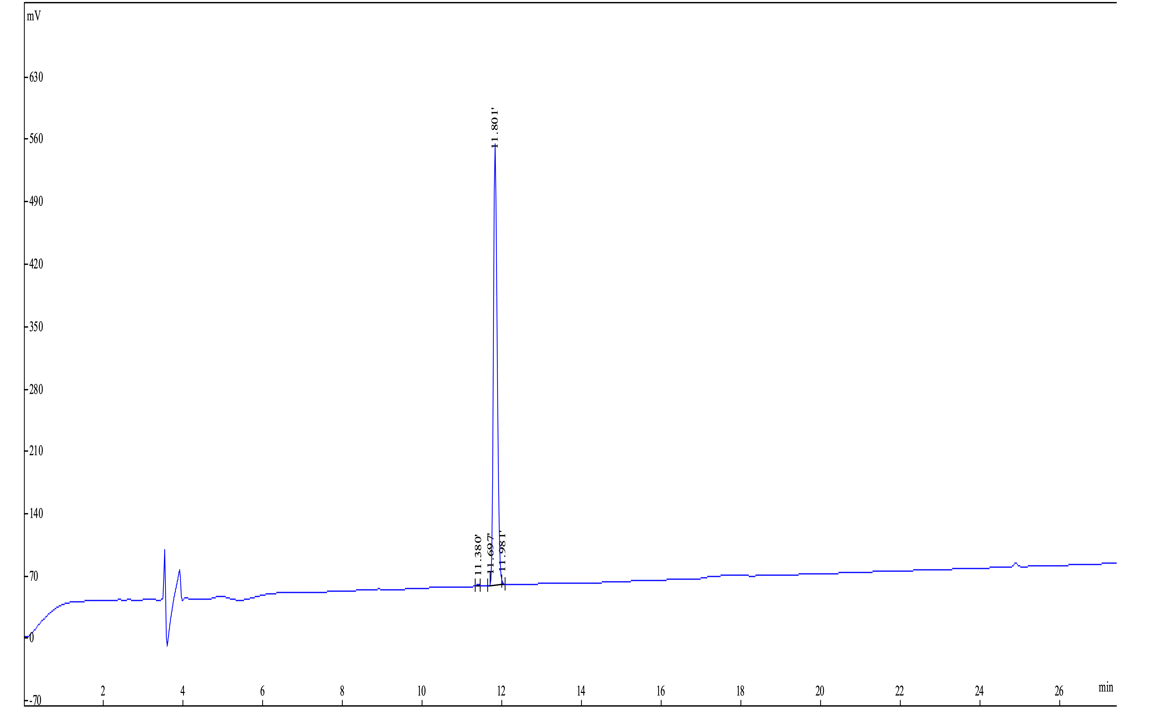


B

**Supplementary Fig. S1**: **Mass spectrometry (A) and HPLC analyses (B) of peptide G4 (VGCARRYCS)** (**Lot #A,** **synthetized and analyzed by Proteogenix).**

RP-HPLC of peptide G4 (Lot #A) was performed using an Agela 100-5C18 (4.6 x 250 mm, 5 µm) column eluted with mobile phase A (0.1% TFA in water) and mobile phase B (0.1% TFA in 100% acetonitrile) using the following chromatographic method: 10% B (0 min), 0−35% B linear-gradient (25 min); flow rate, 1 ml/min. The chromatographic separation was monitored at 220 nm.


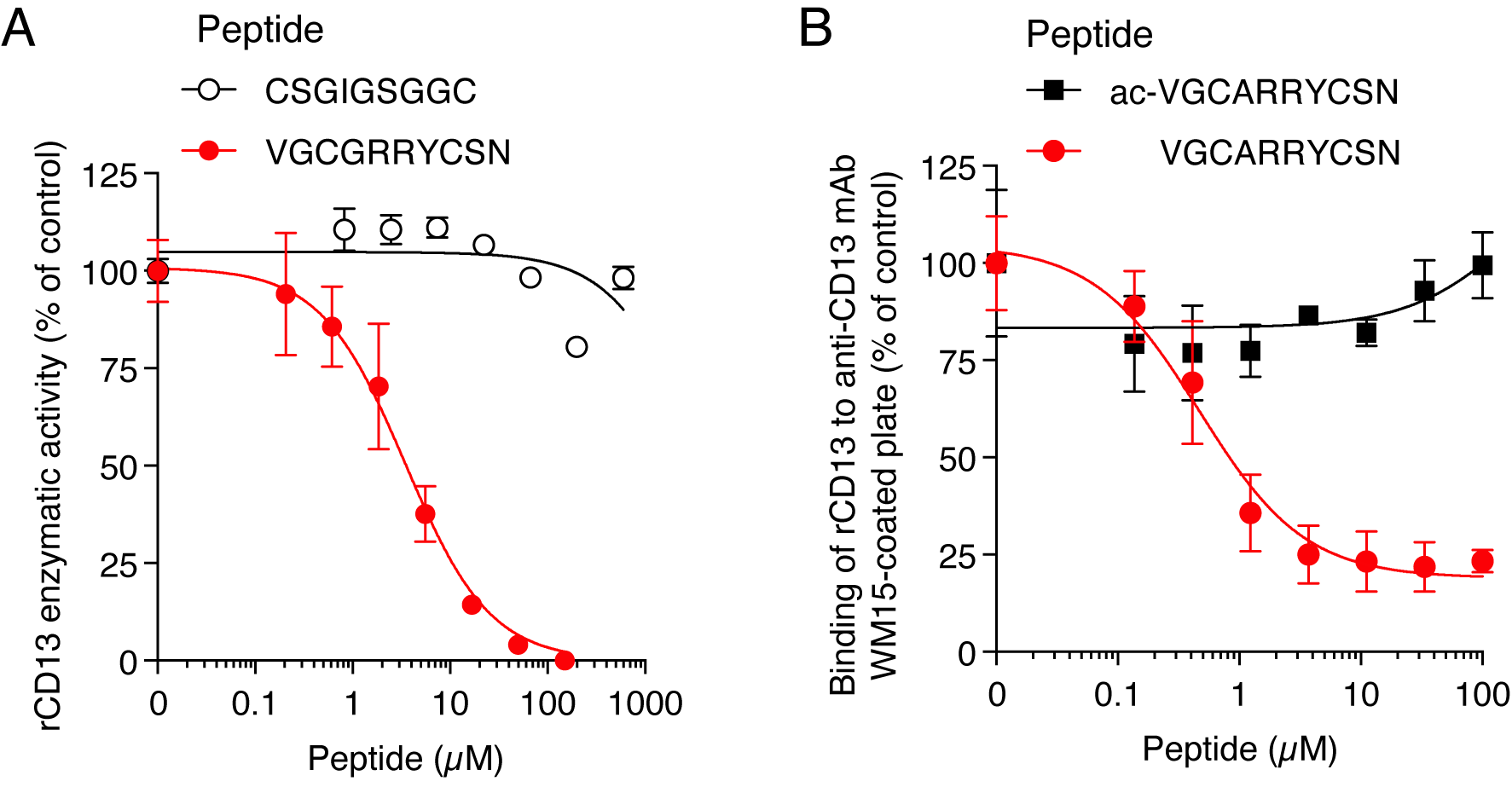


**Supplementary Fig S2.** **Inhibitory activity of VGCGRRYCS on rCD13 enzymatic activity (A) and competitive binding of the anti-CD13 mAb WM15 to rCD13 with VGCARRYCSN or ac-VGCARRYCSN (N-terminal acetylated peptide) (B).**

**A**) VGCGRRYCSN and CSGIGSSGGC (control) peptides, at various concentrations, were mixed with rCD13 (0.2 µg/mL), L-alanine-p-nitroanilide substrate (0.5 mM), in 60 mM potassium phosphate buffer, pH 7.4. The mixtures were left to react for 30 min at 37° C; then the absorbance at 405 nm of each mixture was measured using an ELISA plate reader (Bio-Rad). The IC_50_ of each inhibitor was calculated by non-linear regression analysis of inhibition data using the GraphPad Prism Software (GraphPad Software, Version 9.00, San Diego, CA). The result of one experiment performed in duplicate is shown (mean ± SEM).

**B**) The binding of peptides to rCD13 was assessed by a competitive ELISA based on the use of microtiter plates coated with mAb WM15 (solid-phase). Mixtures of peptides at various concentrations and His-tagged rCD13 in DPBS were added to each well and incubated for 2 h at room temperature. Bound rCD13 was detected with a peroxidase-labelled anti-His-tag antibody, as described (26). The results of one experiment are shown (duplicates, mean ± SEM).

**Supplementary Fig. S3. Proteolytic effect of rCD13 on peptide G4**.


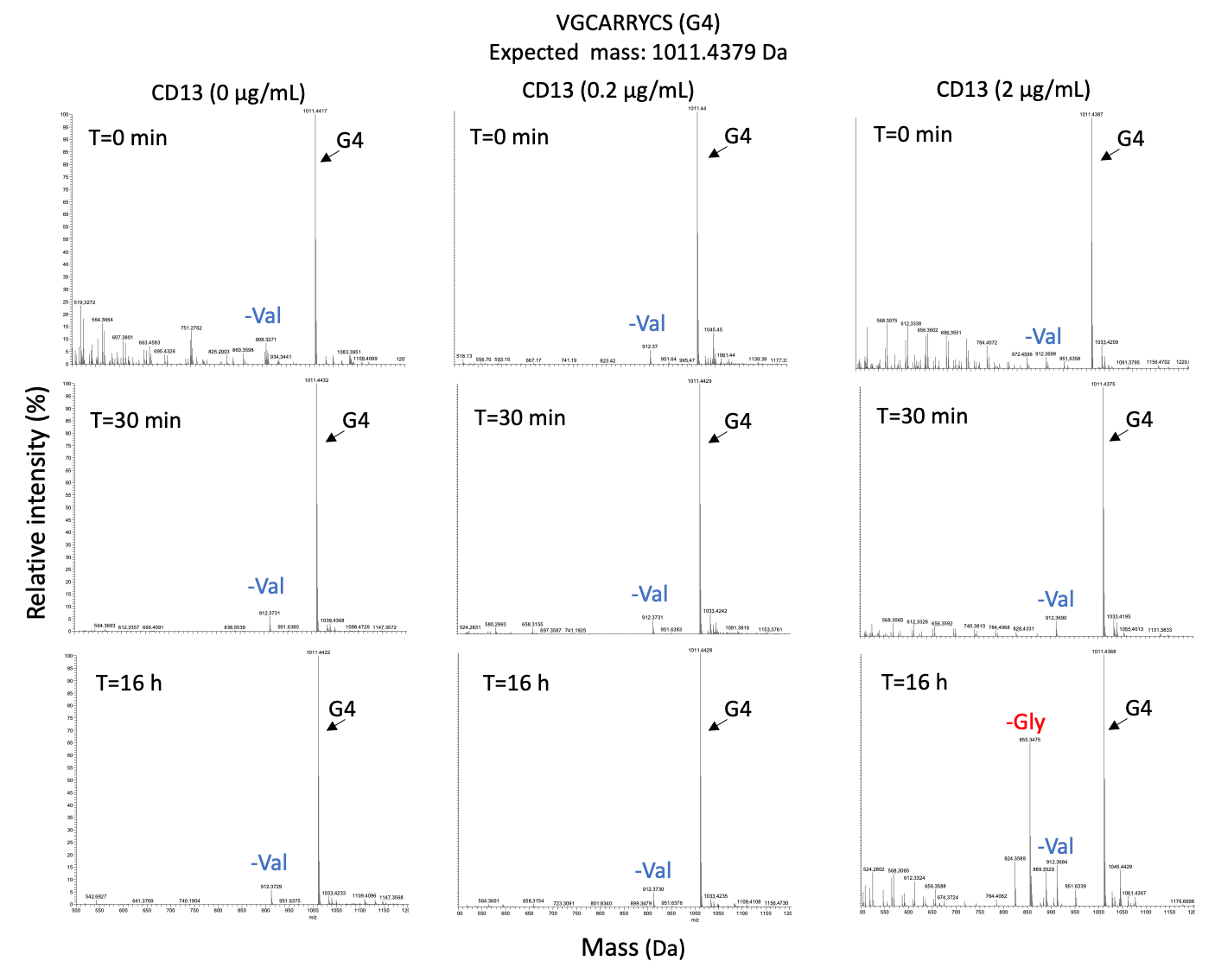


1011.44

1011.4387

1011.4417

912.37

912.3731

912.3729

912.37

912.3731

912.3730

912.3699

912.3690

912.3684

855.3475

1011.4432

1011.4422

1011.4429

1011.4428

1011.4375

1011.4368

0

Peptide G4 (Lot #B, synthetized and analyzed in-house) (10 mM, in 60 mM Tris-HCl, pH 7.4) was incubated with various amounts of rCD13 (0, 0.2 and 2 µg/mL) for 0, 0.5 and 16 h, as indicated, at 37°C. The enzyme activity was stopped by adding 10% acetic acid. Each sample was then desalted using Zip-tip C18 columns (Merck Millipore), dried in a SAVANT centrifuge, resuspended in 8 µl of 10% formic acid, and analyzed by high resolution mass spectrometry, using a Q-Exactive mass spectrometer (Thermo Scientific, Bremen, Germany) equipped with a nano-electrospray ion source (Proxeon Biosystems). Full scan spectra were acquired with the resolution set to 70000 and mass range from 300 to 1500 m/z. The mass spectra were deconvoluted using the Xtract option within the QualBrowser application of the Thermo Xcalibur software v.4.2.47. The presence of GCARRYCS (-Val) in the sample without CD13 is likely an artifact caused by the in-house MS analysis, as suggested by the fact that the analysis of a peptide G4 Lot #A showed the same -Val component when analysed in-house, but not when this product was analysed by Proteogenix with a different methodology (see **Supplementary Fig. S1)**. In any case, considering that the -Val peak (present at time 0 min), increased only after incubation with a large amount of CD13 (2 µg/mL) for a long incubation time (16 h) (together with another peak corresponding to a -Val/-Gly peptide), only this peptide can be considered a degradation product generated by CD13, suggesting that G4 is a poor subtract of CD13.


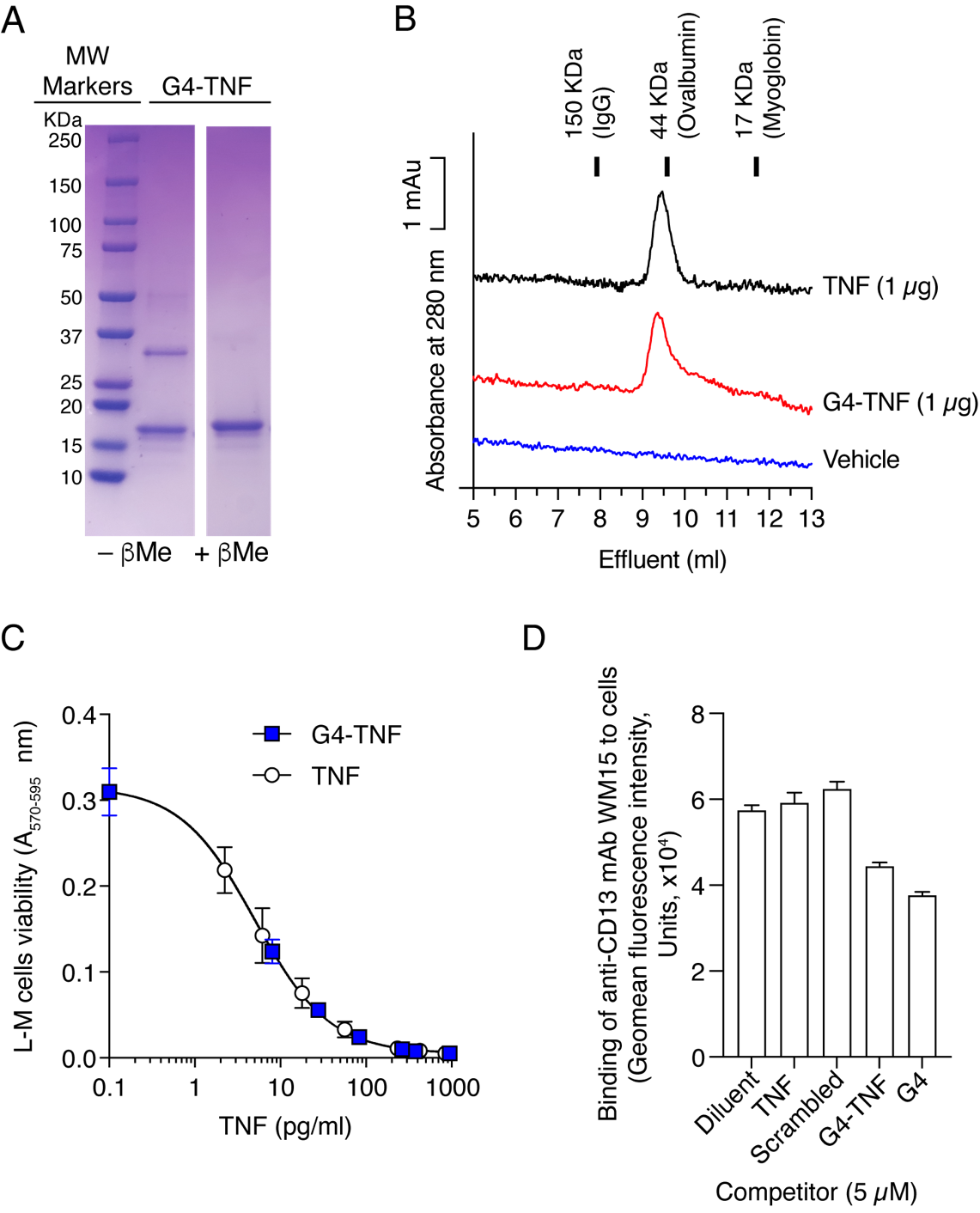


**Supplementary Figure S4. Characterization of purified G4-TNF**

**A)** SDS-PAGE under nonreducing (-βME) or reducing conditions (+βME) of G4-TNF. **B**) Analytical gel-filtration chromatography of 1 µg of TNF or G4-TNF loaded onto a Superdex-75 column. Bars indicate the elution volumes of the molecular weight markers used to calibrate the column, **C**) Cytotoxic effect of G4-TNF and TNF on L-M fibroblasts (each point represents the mean ± SEM of triplicates of one experiment). **D**) Competition of mAb WM15 (anti-CD13) binding to HUVEC cell membrane with G4 and G4-TNF. TNF and a peptide with a scrambled G4 sequence (GCRSNCYRVG) were also used as controls. HUVEC cells (2×10^5^ cells in 100 µL DPBS supplemented with 5% NHS) were seeded and incubated for 60 min at 4°C with mixtures of mAb WM15 (0.13 nM) and G4-TNF, G4, scrambled peptide, or TNF (5 µM). The bound antibody was then detected by flow cytometry analysis, after addition of 2 µg/mL FITC-goat anti-mouse secondary antibody. Cells incubated without the primary antibody served as controls. The binding was assessed by flow cytometry. Three independent experiments were performed. The results of one representative experiment, each performed in duplicate, are shown (mean ± SEM).

**
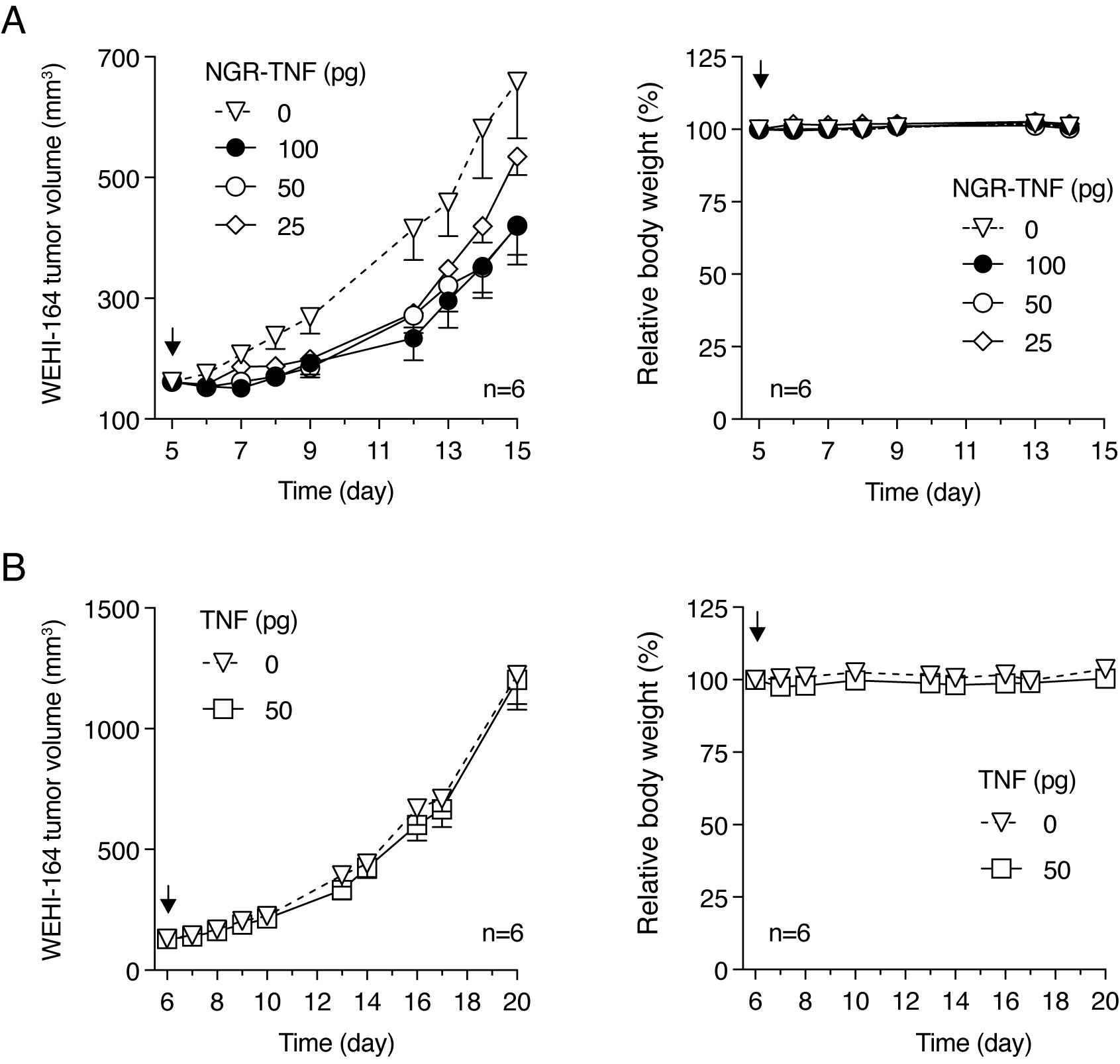
**

**Supplementary Fig. S5. Effect of murine NGR-TNF and TNF on tumor growth and body weight of mice bearing WEHI-164 fibrosarcomas.**

**A)** Mice bearing subcutaneous WEHI-164 tumors (6 mice/groups), were treated 5-6 days after tumor implantation with a single intraperitoneal injection of murine NGR-TNF (0, 25, 50, or 100 pg). Tumor volume and body weight after treatment were monitored daily.

**B)** Effect of murine TNF (50 pg) on tumor growth and body weight. The arrows indicate the time of treatment (mean ± SEM).

**
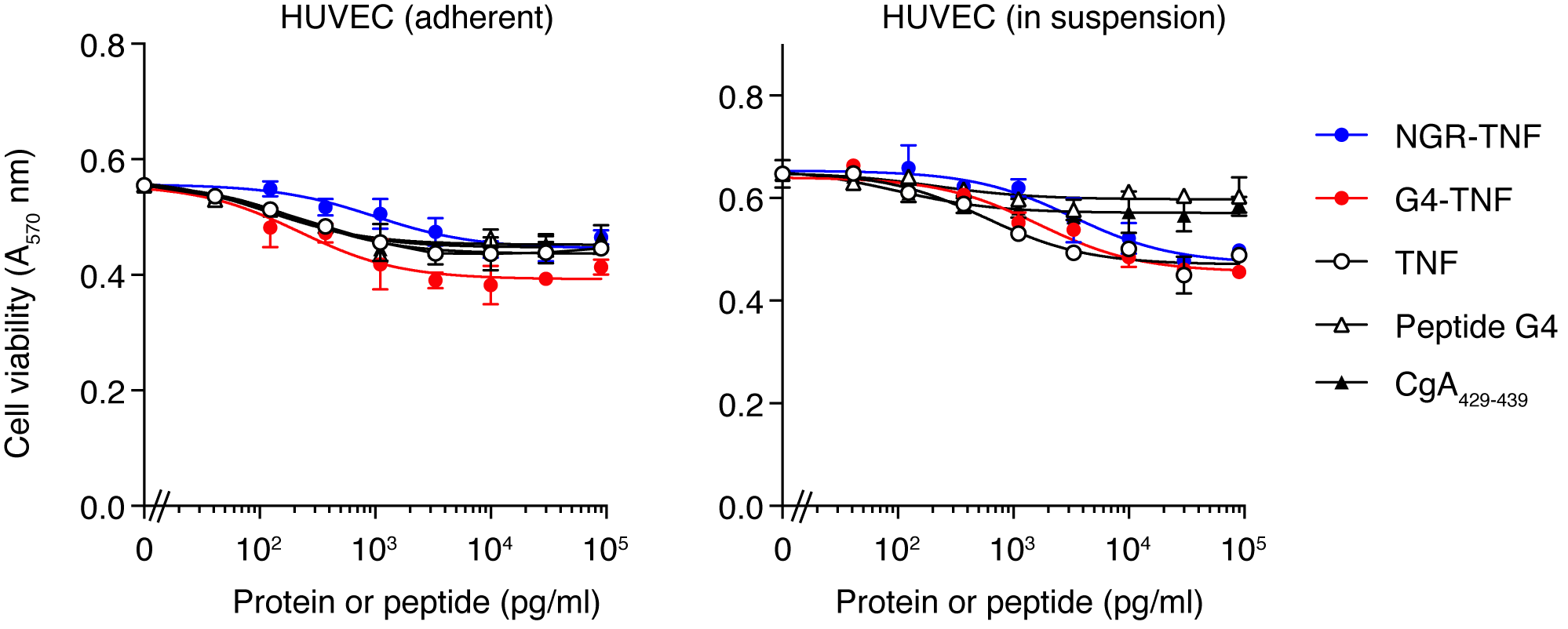
**

**Supplementary Fig. S6. Cytotoxic effects of G4-TNF, NGR-TNF, TNF, G4 and CgA429-439 on adherent HUVEC cells and cells in suspension.**

The cytotoxic assay with adherent cells was performed as follows: HUVEC (LONZA, cod #LO191027, Batch #18TL232828) were cultured in EGM2-Bullet KIT (LONZA cod #LOCC-3162), detached with a trypsin-EDTA solution and plated in 96-well flat-bottom plate for cell culture (30000 cells/well, 100 µL/well) in complete medium and incubated overnight at 37°C, 5% CO_2_. One hundred µL of G4-TNF, NGR-TNF or TNF solutions in complete medium were then added to each well and left to incubate for 24 h at 37°C, 5% CO_2_. Ten µL aliquots of MTT solution, freshly prepared at 5 mg/mL in PBS, were then added to the wells and left to incubate for 3 h at 37°C, 5% CO2. The supernatant was removed using a Pasteur pipet connected to a vacuum pump, and 200 µL of dimethyl sulfoxide was then added to each well to dissolve the formazan crystals. The absorbance at 570 nm was then read using a microtiter plate reader.

The cytotoxicity assay with cell in suspension was carried out following the same protocol except that the overnight incubation of the microtiter plate after cell plating was omitted.
